# Hypoxia-induced long noncoding RNA NR2F1-AS1 maintains pancreatic cancer proliferation, migration, and invasion by activating the NR2F1/AKT/mTOR axis

**DOI:** 10.1038/s41419-022-04669-0

**Published:** 2022-03-14

**Authors:** Yanqing Liu, Shiyu Chen, Kun Cai, Dijie Zheng, Changhao Zhu, Lin Li, Feiqing Wang, Zhiwei He, Chao Yu, Chengyi Sun

**Affiliations:** 1 College of Basic Medicine, Guizhou Medical University, Guiyang, Guizhou China; 2 The First Affiliated Hospital of Guizhou University of Traditional Chinese Medicine, Guiyang, Guizhou China; 3 Department of Translational Medicine, College of Clinical Medicine, Guizhou Medical University, Guiyang, Guizhou China; 4 Department of Hepatic-Biliary-Pancreatic Surgery, The Affiliated Hospital of Guizhou Medical University, No. 9, Beijing Road, Guiyang, Guizhou Province 550000 China; 5 College of Clinical Medicine, Guizhou Medical University, Guiyang, Guizhou China

**Keywords:** Pancreatic cancer, Cancer microenvironment

## Abstract

Accumulating evidence has demonstrated the essential role of long noncoding RNAs (lncRNAs) in various types of human cancer, including pancreatic cancer (PC). However, the functions and regulatory mechanisms of nuclear receptor subfamily 2 group F member 1 antisense RNA 1 (NR2F1-AS1) that are responsible for its role in the malignant progression of PC cells remains to be investigated. In this study, the biological effects of NR2F1-AS1 and NR2F1 in PC were investigated by in vitro and in vivo experiments. The mechanisms of NR2F1-AS1 were monitored by bioinformatic predictive analysis and confirmatory experiments. Our results indicated that NR2F1-AS1 was overexpressed and positively correlated with poor survival in PC. Depletion of NR2F1-AS1 restrained PC cell proliferation, migration, invasion, and suppressed xenograft tumor growth and metastasis in vitro and in vivo. Mechanistic experiments suggested that NR2F1-AS1 positively regulated the neighboring *NR2F1* gene, which subsequently activated AKT/mTOR signaling, resulting in the upregulation of hypoxia-inducible factor-1α (HIF-1α). Further investigations elucidated that NR2F1-AS1 expression was transcriptionally regulated by HIF-1α under hypoxia. These findings demonstrated that hypoxia-induced NR2F1-AS1 expression directly increased NR2F1 levels to promote PC cell proliferation, migration, and invasion by activating AKT/mTOR signaling. Together, these findings suggest that NR2F1-AS1 could be a prospective therapeutic target for PC.

## Introduction

Pancreatic cancer (PC) is among the most fatal malignancies, ranking among the top ten causes of all cancer-associated mortality, with early local infiltration and distant metastasis being the predominant reasons for death [1]. The vast majority of PC patients already have advanced-staged disease upon diagnosis, which is an indicator of poor prognosis [2]. At later stages, tumor cells are more likely to have migrated to nerves, blood vessels, and distant organs, and local tissue infiltration negatively impacts survival outcomes in PC patients [3]. Nevertheless, the underlying molecular mechanisms that trigger PC invasion and metastasis remain unclear. Therefore, exploring these molecular mechanisms is momentous for identifying prognostic biomarkers and developing treatments for advanced PC.

Recently, long noncoding RNAs (lncRNAs) have been revealed to be pivotal moderators of cancer evolvement due to their multitudinous functions and mechanisms [4]. LncRNAs participate in multiple biological processes through extensive mechanisms, such as cell proliferation, differentiation, apoptosis, invasion, and maintenance of stem cell pluripotency [5]. Numerous studies have manifested that lncRNAs are crucial molecules involved in gene regulation, including in the recruitment of chromatin-modifying complexes and transcriptional co-regulators [6]. Specifically, accumulating reports have demonstrated that numerous differentially regulated lncRNAs are involved in malignant progression and correlate with prognosis in PC patients [7]. For instance, TP53TG1 acts as an oncogene that accelerates PC proliferation, migration, and invasion by interplaying with miR-96 to regulate KRAS network expression [8]. LncRNA-HOTAIR is upregulated in PC and participates as an oncogene in cancer progression and chemotherapy resistance through multiple mechanisms [9]. Studies have indicated that lncRNAs have extensive functions that mediate PC progression, and thus could serve as either prognostic markers or therapeutic targets. Therefore, shedding light on the molecular mechanism of lncRNAs is of great significance for future prognostic and therapeutic modalities in PC.

Hypoxia is a peculiarity of the tumor microenvironment that drives a series of malignant phenotypes such as excessive proliferation, angiogenesis, metastasis, metabolic reprogramming, and immune escape [10]. Hypoxia-inducible factor (HIF)-1α and -2α are two key factors that mediate the responses of cells to hypoxia [11]. In addition, hypoxia-induced lncRNAs have been found to have important impacts on the HIF pathway, tumorigenesis, and cancer progression [12]. For instance, lncRNA metastasis-associated protein 2 transcriptional regulator (MTA2TR) is elevated and facilitates PC progression through a mechanism that is related to the reciprocal regulation of HIF-1α [13]. Another recent study revealed that NR2F1-AS1 was increased under hypoxia and contributed to hypoxia-triggered glycolysis and migration via regulating the miR-140/HK2 pathway in hepatocellular carcinoma (HCC) [14]. However, how lncRNAs interact with hypoxia moderators during PC progression has remained largely elusive.

Nuclear receptor subfamily 2 group F member 1 antisense RNA 1 (NR2F1-AS1) is a natural antisense transcript (NAT) lncRNA that has been reported to serve as a carcinogenic factor in several types of malignant tumors, including HCC [14, 15], osteosarcoma [16], endometrial cancer [17], colorectal cancer [18], and breast cancer [19, 20]. In this work, we performed bioinformatic analysis to screen lncRNAs that are differentially expressed in PC and identified NR2F1-AS1 as an upregulated lncRNA that was associated with poor prognosis in PC patients. Furthermore, chromatin immunoprecipitation (ChIP)-sequencing and The Cancer Genome Atlas (TCGA) data followed by bioinformatic analysis revealed that NR2F1-AS1 was associated with hypoxia and metastasis. We show that NR2F1-AS1 enhanced the expression of its neighbor gene *NR2F1*, which subsequently activates the AKT/mTOR pathway to facilitate PC progression. Moreover, we further elucidated that NR2F1-AS1 was transcriptionally induced by HIF-1α under hypoxic conditions, thereby promoting the malignant progression of hypoxic PC cells. Therefore, our results implicate that NR2F1-AS1 contributes to PC development through *NR2F1* and the AKT/mTOR pathway, which involved in a hypoxic microenvironment, providing a potential prognostic biomarker and therapeutic target for PC.

## Materials and methods

### Patients and clinical samples

Ninety paired PC and matched paracancerous tissue samples were collected from patients who were diagnosed with PC and underwent resection at the Affiliated Hospital of Guizhou Medical University between 2018 and 2020. All PC tissues were confirmed by the histopathological reports. The collected tissues were quickly stored in a −80 °C freezer. This study was approved by the Institutional Review Board of the Affiliated Hospital of Guizhou Medical University before sample collection (IRB: No. 2018–017), and all patients had provided written informed consent.

### Cell lines and cell culture

The human PC cell lines BxPC-3, Capan-2, CFPAC-1, SW1990, MIA PaCa-2, and PANC-1 were purchased from the American Type Culture Collection (Manassas, VA, USA), while the human pancreatic duct epithelial (HPDE) cells were purchased from Shanghai Cell Bank of the Chinese Academy of Sciences (Shanghai, China). According to ATCC suggestions, the cell lines were cultured in complete growth medium. Cells were cultured at 37 °C in a humidified atmosphere of 5% CO_2_. The indicated cells were also cultured in 1% O_2_, 5% CO_2_, and 94% N_2_ or treated with 100 μM CoCl_2_ [21] (Sigma-Aldrich, St. Louis, MO, USA) to induce hypoxia.

### In situ hybridization (ISH)

ISH was performed using the RNA ISH Kit according to the manufacturer’s direction (Exiqon, Germany). Briefly, the tissue chips were baked at 63 °C for 1 h, and then xylene was used for dewaxing, and reagent grade dilute ethanol was used for rehydration. The slides were then digested with 5 μg/mL protease K at 37 °C for 2 min, dehydrated, and then incubated with 20 nM double (5′- and 3′-) DIG-labeled oligonucleotide ISH probe for NR2F1-AS1 (Supplementary Table S1) in hybridization buffer at 50 °C for 1 h, followed by washing and incubation with anti-DIG-AP Fab fragment antibody overnight at 4 °C. Finally, BCIP/NBT Substrate developing solution was applied to the sections for signal detection and incubated at 37 °C for 30 min. Images were captured using an Aperio ImageScope system.

### RNA fluorescence ISH (RNA-FISH)

RNA-FISH was performed using the Ribo™ lncRNA FISH Probe Mix (Red) (Guangzhou RiboBio Co., Ltd., Guangdong, China) according to the manufacturer’s instructions and suggestions. The indicated cells were seeded into 24-well plates at 6 × 10^4^ cells/well and cultured until they reached 60 to 70% confluence. Cells were then fixed in 4% paraformaldehyde and treated with precooled PBS containing 0.5% Triton X-100 at 4 °C for 5 min. After being incubated with prehybridization and hybridization solutions, respectively, the cells were incubated with the lncRNA NR2F1-AS1 FISH probe mix (20 μM) at 37 °C overnight. Then the cells were washed with SSC hybrid washing buffer solution for 5 min. After being washed with PBST, an anti-fluorescence quencher was added, and then the cells were observed using a fluorescence microscope (Olympus, Tokyo, Japan).

### Cell transfection and infection

Small interfering RNAs (siRNAs) and appropriate negative control (NC) were obtained from RiboBio and transfected into cells using riboFECT™ CP Buffer (RiboBio) according to the manufacturer’s protocol. Short hairpin RNAs (shRNAs) containing NR2F1-AS1 mimics, NC mimics, sh-Control, sh-NR2F1-AS1#1, and NR2F1-AS1#2 lentivirus were provided by Shanghai Genechem Co., Ltd. (Shanghai, China). Cells were infected with shRNAs or mimics using infection booster solutions Hitrans G, A, or P according to the manufacturer’s instructions. After 12–16 h, the medium was replaced with complete medium. Total RNA and protein were extracted at 24 or 48 h post-infection for downstream analyses. The siRNA sequences used in this study are listed in Supplementary Table S2.

### Immunofluorescence (IF) staining

Cells were fixed in 4% formaldehyde and washed with 1× PBST for 30 min. The cells were blocked with 5% bovine serum albumin (BSA) for 2 h, and then incubated with primary anti-E-cadherin, anti-Vimentin, or anti-NR2F1 antibodies (Supplementary Table S3) at 4 °C for 18–24 h. After being washed with 1× PBST, fluorescent FITC-conjugated AffiniPure goat anti-rabbit IgG (H + L) secondary antibody was added and incubated for 45 min. The cells were again washed and incubated with DAPI (Invitrogen, Carlsbad, CA, USA) for nuclear staining. The cells were visualized using a fluorescence microscope (Olympus).

### Xenograft models experiment

For subcutaneous xenograft PC models, PANC-1 or MIA PaCa-2 (5 × 10^6^ cells/mouse) cells that had been transfected with sh-Control, sh-NR2F1-AS1#1, or sh-NR2F1-AS1#2 were subcutaneously injected into the right flank of female nude BALB/c mice (4–6-weeks-old, HFK Bio-Technology Co., Ltd, Beijing, China). To establish the metastasis model, 5 × 10^6^ PANC-1 or MIA PaCa-2 cells stably expressing sh-Control or shRNAs were transplanted into the spleen through the splenic envelope. BALB/c nude mice were then randomly divided into three groups with five mice per group. Tumor volumes of the mice were monitored every week after implantation. Tumor growth was detected weekly by bioluminescent imaging with a Xenogen IVIS (PerkinElmer, Waltham, MA, USA). Five weeks after implantation, the mice were sacrificed, and the xenograft tumors were collected and measured. All animal experiments were carried out in accordance with the principles and procedures authorized by the Animal Experimental Ethics Committee of Guizhou Medical University.

### Dual-luciferase reporter assay

Using the hypoxia response elements (HREs) in the NR2F1-AS1 promoter region, we constructed a wild-type (WT) and mutated (MUT) sequence containing two putative HIF-1α binding sequences into pGL3-based vectors (RiboBio). Luciferase reporter plasmid pGL3-based vectors expressing NR2F1-AS1-WT or MUT were co-transfected into MIA PaCa-2 and PANC-1 cells in 96-well plates with siNC or siHIF-1α. After 24 h, cells were cultured under normoxia or hypoxia for an additional 24 h. Cell lysates were harvested, and firefly and Renilla luciferase activities were measured using the dual-luciferase reporter system kit (Beyotime, Shanghai, China) on a microplate reader according to the manufacturer’s suggestions and protocols. The Renilla luciferase internal control was used to normalize luciferase activity.

### Statistical analyses

All results are shown as mean ± standard deviation (SD) and represent the results of three independent experiments. Statistical analyses were performed using GraphPad Prism 8.0 (GraphPad Software, Inc., San Diego, CA, USA) and SPSS 22.0 software (IBM Corp., Armonk, NY, USA). Fisher’s exact test (two-sided) or the Chi-square test were used to analyze correlations between NR2F1-AS1 expression levels and clinicopathological features of PC patients. Kaplan–Meier curves and the log-rank test were used to analyze overall survival rates. Pearson’s correlation analysis was adopted for correlation analysis between genes. Student’s *t* test or one-way ANOVA were used for statistical comparisons between different groups. The data met all assumptions of the tests, respectively. A *P* value < 0.05 was used to indicate significant differences (*P < 0.05, **P < 0.01, and ***P < 0.001).

## Results

### NR2F1-AS1 expression is enhanced in PC tissue and cell lines

**Fig. 1:**
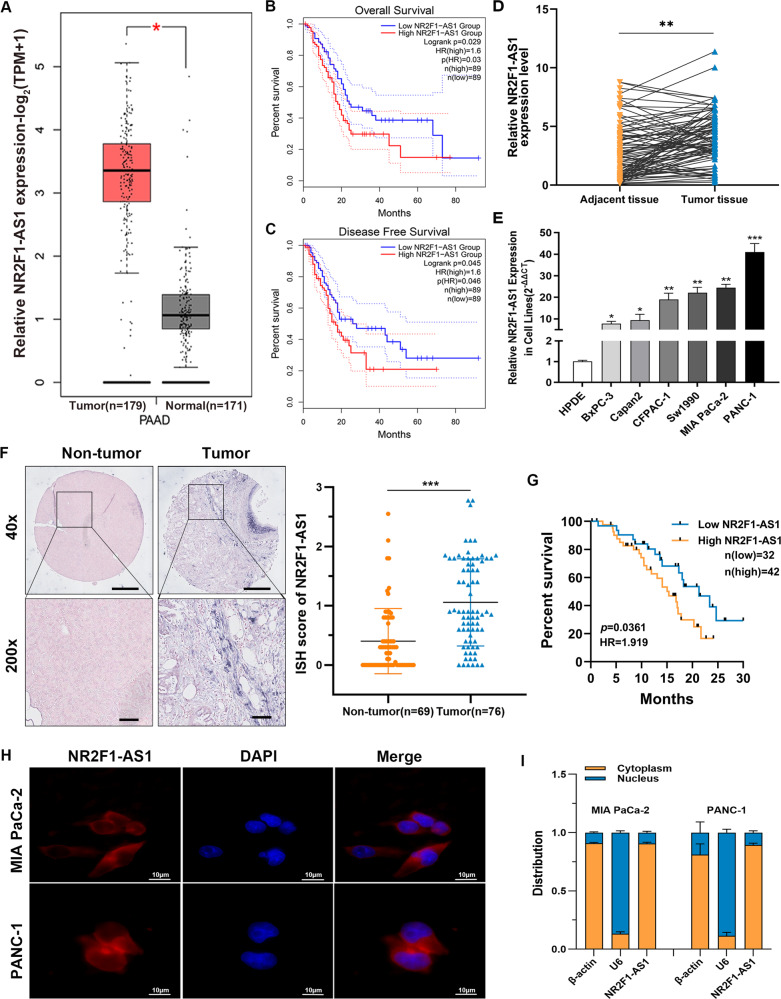
NR2F1-AS1 expression is increased in PC tissues and cell lines. **A** Bioinformatics analysis of NR2F1-AS1 expression using TCGA-PAAD database on GEPIA (http://gepia2.cancer-pku.cn/#index) (T, tumor; N, normal). **B**, **C** Kaplan–Meier curves showing overall survival (OS) (**B**) and disease-free survival (DFS) (**C**) in PC patients stratified according to NR2F1-AS1 expression in TCGA-PAAD cohort. **D** qRT-PCR analysis of NR2F1-AS1 expression in 90-paired PC and matched adjacent tissues. **E** qRT-PCR analysis of NR2F1-AS1 expression in six PC cell lines and normal HPDE cells. **F** Analysis of NR2F1-AS1 expression in PC and non-tumor tissues by ISH; scale bar = 500 μm. **G** Survival analysis according to NR2F1-AS1 expression among 74 PC patients via Kaplan–Meier curves compared with the log-rank test. **H** FISH to detect the subcellular localization of NR2F1-AS1 (red) in MIA PaCa-2 and PANC-1 cells. Nuclei were counterstained with DAPI; scale bar = 10 μm. **I** qRT-PCR analysis of the localization and expression levels of NR2F1-AS1 in the cytoplasm and nucleus of MIA PaCa-2 and PANC-1 cells. Data are expressed as mean ± SD; *P < 0.05, **P < 0.01, ***P < 0.001.

According to TCGA data accessed via the GEPIA website, we identified NR2F1-AS1 to have markedly higher expression in pancreatic adenocarcinoma (PAAD) samples (Fig. 1A). Moreover, high NR2F1-AS1 expression was also verified in several other PC and metastatic tumor Gene Expression Omnibus (GEO) databases (GSE15471, GSE16515, GSE58561, GSE91035, and GSE63124) (Supplementary Fig. S1A–H). Furthermore, using TCGA data, Kaplan–Meier analysis implied that patients with high NR2F1-AS1 expression had a poor prognosis (Fig. 1B, C). Nevertheless, the results from TCGA data, as shown in Supplementary Fig. S1I–L, indicated that NR2F1-AS1 expression was not correlated with lymph node metastasis, tumor stage, metastasis stage, or histologic grade. To validate these bioinformatic analysis results, increased NR2F1-AS1 expression was confirmed by quantitative real-time polymerase chain reaction (qRT-PCR) and ISH in PC tissues and cell lines (Fig. 1D–F). Kaplan–Meier analysis showed that high NR2F1-AS1 expression in PC patients was associated with a lower survival rate (Fig. 1G). On the basis of the clinicopathologic characteristics of PC patients, we found that high NR2F1-AS1 expression was associated with large tumor size and perineural invasion (Supplementary Table S4). In addition, we performed RNA-FISH and RNA extraction from specific cell fractions to confirm the localization of NR2F1-AS1. These results revealed that NR2F1-AS1 was mostly cytoplasmic in PC cells (Fig. 1H, I). Collectively, these results suggested that NR2F1-AS1 was abnormally elevated in PC cells and might be a potential biomarker of pancreatic malignancy.

### NR2F1-AS1 promotes the proliferation, migration, and invasion of PC cells in vitro

**Fig. 2:**
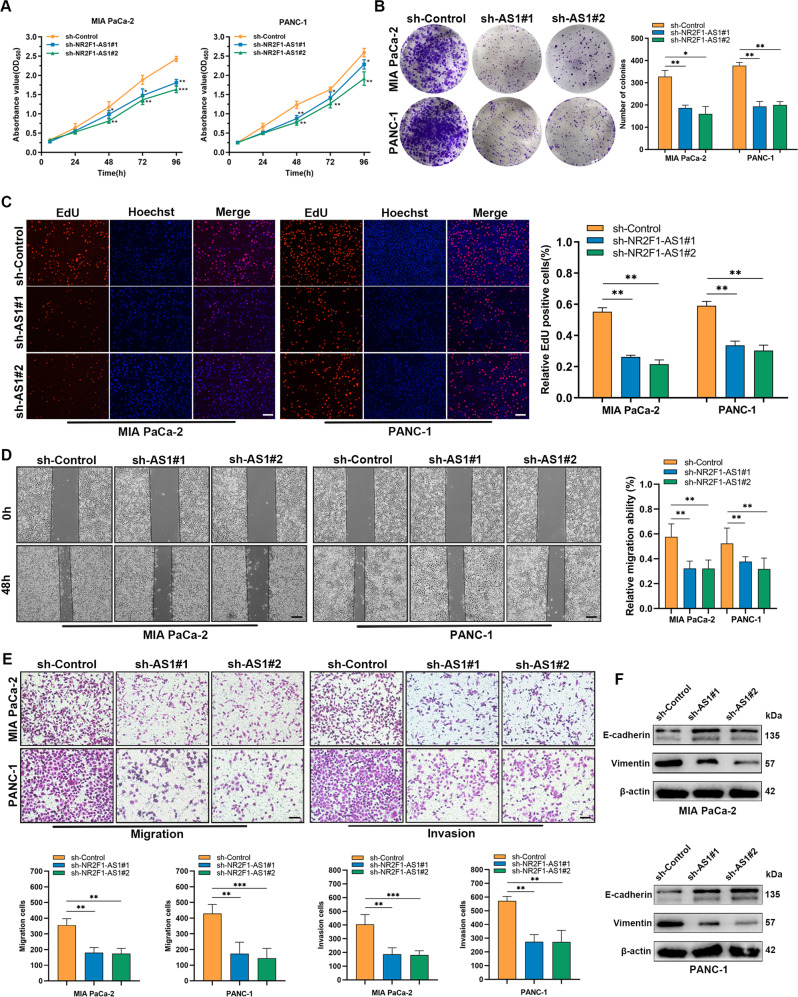
NR2F1-AS1 promotes the proliferation of PC cells in vitro. **A** CCK-8 assays were performed to examine the proliferation of NR2F1-AS1-depleted MIA PaCa-2 and PANC-1 cells. **B** Representative images of colony-formation assays in NR2F1-AS1-knockdown and control MIA PaCa-2 and PANC-1 cells. **C** EdU proliferation assays in NR2F1-AS1-knockdown and control MIA PaCa-2 and PANC-1 cells. **D** Wound-healing assays in NR2F1-AS1-knockdown and control MIA PaCa-2 and PANC-1 cells. **E** Transwell assays were used to evaluate the migration and invasion abilities of NR2F1-AS1-knockdown and control MIA PaCa-2 and PANC-1 cells. **F** Western blot analysis of epithelial-to-mesenchymal transition markers in NR2F1-AS1-knockdown and control PC cells. Scale bar = 200 μm. Data are expressed as mean ± SD. All experiments were repeated three times independently. *P < 0.05, **P < 0.01, ***P < 0.001.

To elucidate the function of NR2F1-AS1 on cell biological behaviors, we generated stable MIA PaCa-2 and PANC-1 cell lines with lentiviral-mediated NR2F1-AS1 silencing (sh-NR2F1-AS1#1 and sh-NR2F1-AS1#2) or control (sh-Control) with verified the knockdown efficiency (Supplementary Fig. S2A). Functional assays using these cell lines revealed that NR2F1-AS1 depletion remarkably inhibited PC cell proliferation (Fig. 2A–C). Moreover, subsequent overexpression of NR2F1-AS1 could salvage the impaired proliferation ability of NR2F1-AS1-downregulated cells (Supplementary Fig. S3A–C). Next, wound-healing and transwell assays demonstrated that NR2F1-AS1 knockdown suppressed the migration and invasion capacities of PC cells (Fig. 2D, E); both phenotypes were also rescued by re-expressing NR2F1-AS1 (Supplementary Fig. S3D–G). Epithelial-to-mesenchymal transition (EMT) is a crucial feature of tumor invasion and metastasis. Therefore, western blot and IF assays were used to examine EMT markers, including E-cadherin and Vimentin. The results demonstrated that silencing NR2F1-AS1 facilitated E-cadherin expression but decreased Vimentin expression (Fig. 2F and Supplementary Fig. S2B, C). Taken together, these results demonstrated that silencing NR2F1-AS1 weakened the tumorigenicity of PC cells in vitro.

### Depletion of NR2F1-AS1 suppressed PC cell proliferation and metastasis in vivo

**Fig. 3:**
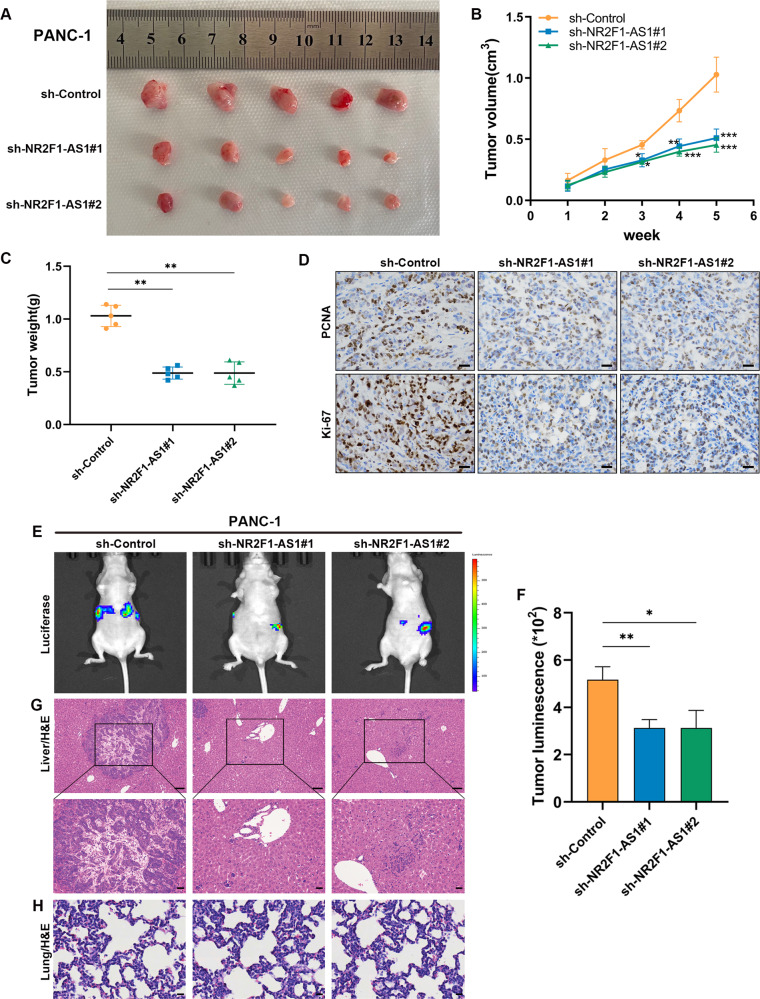
Silencing of NR2F1-AS1 inhibits tumor formation in nude mice. **A** Image of xenograft tumors resected from nude mice-carrying PANC-1 cells that transduced with lentiviruses encoding sh-Control, sh-NR2F1-AS1#1, or sh-NR2F1-AS1#2 (*n* = 5). **B** Growth curves of the subcutaneous tumor were made from weekly measurements of tumor volume. **C** Weights of the subcutaneous tumors in each group were measured after 5 weeks when the mice were sacrificed. **D** Representative images of IHC staining for Ki-67 and PCNA in the tumor sections from sh-Control, sh-NR2F1-AS1#1, or sh-NR2F1-AS1#2 xenografts; scale bar = 100 μm. **E**–**H** Representative fluorescent images of microscopic metastatic nodules after 5 weeks (*n* = 5), and liver (**G**) and lung (**H**) tissues stained with hematoxylin and eosin (H&E); scale bar = 20 μm. Data are expressed as mean ± SD. *P < 0.05, **P < 0.01, ***P < 0.001.

To further explore whether NR2F1-AS1 affected the proliferation of PC cells in vivo, PANC-1 and MIA PaCa-2 cells stably transfected with a lentiviral vector containing sh-NR2F1-AS1#1, sh-NR2F1-AS1#2, or sh-Control were inoculated into nude mice. Five weeks after implantation, the mean volume and weight of tumors in the NR2F1-AS1-knockdown groups were substantially lower than those from the control group (Fig. 3A–C and Supplementary Fig. S4A–C). Immunohistochemistry (IHC) results showed a visible decrease in PCNA and Ki-67 expression in xenograft tumor tissues from the NR2F1-AS1-knockdown groups compared with control tumors (Fig. 3D and Supplementary Fig. S4D). Moreover, the metastatic model indicated that fluorescence intensity was dramatically decreased in livers of mice injected with NR2F1-AS1 knockdown cells compared with in the control group, and histological examination verified the presence or absence of liver and lung metastases in the groups (Fig. 3E–H and Supplementary Fig. S4E–H). These results indicated that depletion of NR2F1-AS1 suppressed the growth and metastasis of PC cells in vivo, which further validated the carcinogenesis of NR2F1-AS1 in PC progression.

### *NR2F1* is a target of NR2F1-AS1 and is positively regulated by NR2F1-AS1

**Fig. 4:**
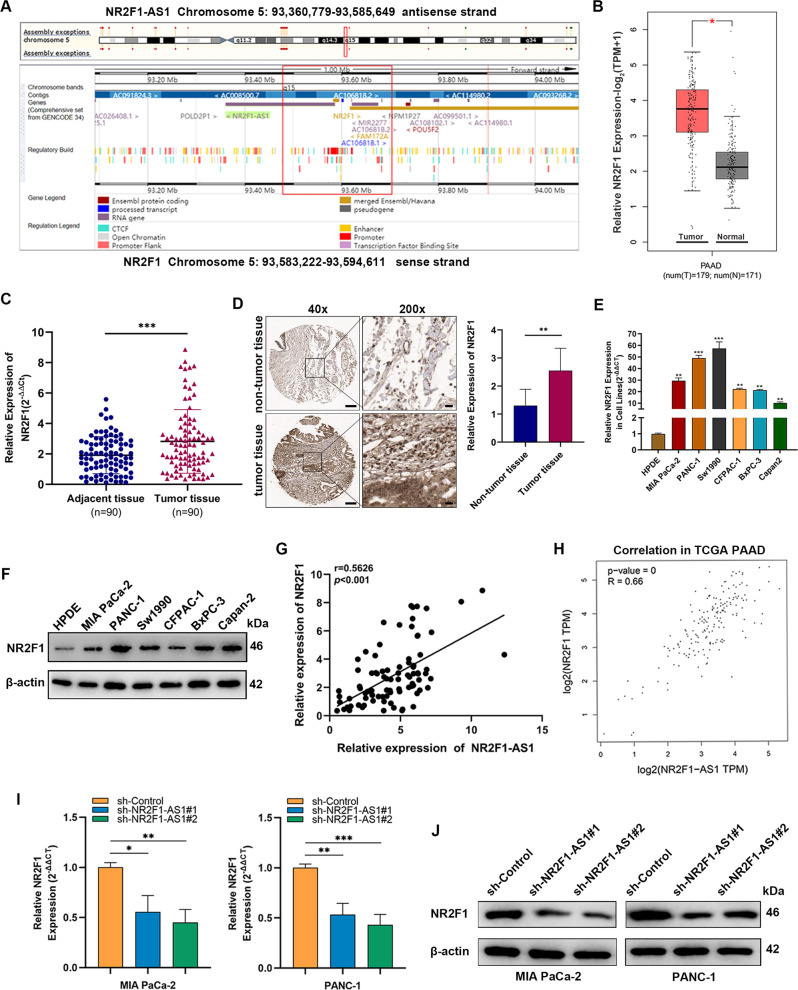
NR2F1 is a target of NR2F1-AS1 and is positively regulated by NR2F1-AS1. **A** Bioinformatics analysis of the correlation between the genomic locations of NR2F1-AS1 and *NR2F1*. **B** The expression level of *NR2F1* in the TCGA-PAAD database (T, tumor; N, normal). **C** NR2F1 expression in PC and matched adjacent tissues (*n* = 90) was detected by qRT-PCR. **D** IHC analysis of NR2F1 expression in PC and adjacent tissues using tissue microarrays; scale bar = 500 μm. **E, F** qRT-PCR (**E**) and western blot (**F**) results showing NR2F1 expression in six PC cell lines compared with the HPDE cell line. **G** Correlation between NR2F1-AS1 and *NR2F1* expression in PC tissues. **H** Correlation between *NR2F1* and NR2F1-AS1 expression levels in the TCGA-PAAD dataset. **I**, **J** qRT-PCR (**I**) and western blot (**J**) results for NR2F1 expression in PANC-1 and MIA PaCa-2 cells after introducing NR2F1-AS1-knockdown constructs (sh-NR2F1-AS1#1 and sh-NR2F1-AS1#2) or the negative control (sh-Control) by lentiviral infection. Data are expressed as mean ± SD. *P < 0.05, **P < 0.01, ***P < 0.001.

Previous reports have revealed that antisense lncRNAs can mediate the expression and chromatin state of genes nearby their own transcription site (in-cis) [22]. Bioinformatics analysis (http://asia.ensembl.org/index.html) showed that NR2F1-AS1 was adjacent to the *NR2F1* gene, located on the antisense strand of NR2F1 (Fig. 4A). Analysis of the GEO and TCGA databases also demonstrated that *NR2F1* was overexpressed in PC tissues (Fig. 4B and Supplementary Fig. S5A, B), which was confirmed by qRT-PCR, IHC, and western blot assays (Fig. 4C–F and Supplementary Fig. S5C). To further clarify the correlation between NR2F1-AS1 and *NR2F1*, Pearson’s analysis indicated that NR2F1-AS1 and *NR2F1* expression levels showed a positive correlation in PC tissues (Fig. 4G, H and Supplementary Fig. S5D, E). Accordingly, qRT-PCR, western blot, and IF assays illustrated that *NR2F1* mRNA and protein expression were dramatically decreased in NR2F1-AS1-knockdown cells (Fig. 4I, J and Supplementary Fig. S5F, G). Taken together, these results suggested that NR2F1-AS1 plays a critical role in regulating the expression of its sense homologous gene, *NR2F1*.

### Silencing *NR2F1* attenuates the proliferation, migration, and invasion of PC cells

**Fig. 5:**
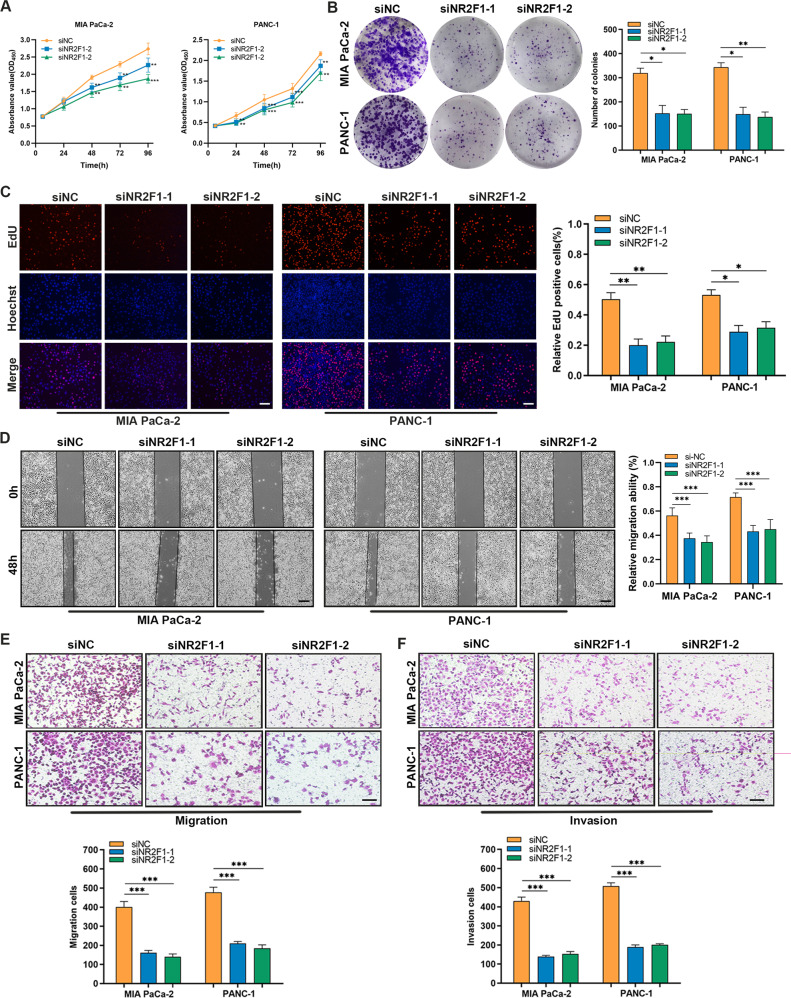
Silencing NR2F1 decreases the proliferation, migration, and invasion capacities of PC cells. **A** CCK-8 assays in the siNR2F1–1, siNR2F1–2, and siNC groups of PANC-1 and MIA PaCa-2 cells. **B** Colony-formation assays in the NR2F1-silenced and NC groups of PANC-1 and MIA PaCa-2 cells. **C** EdU assays in the NR2F1-silenced and NC groups of PANC-1 and MIA PaCa-2 cells. **D** Wound-healing assay in the NR2F1-silenced and NC groups of PANC-1 and MIA PaCa-2 cells. **E**,**F** Transwell assays showing the migration (**E**) and invasion (**F**) abilities of the NR2F1-silenced and NC groups of PANC-1 and MIA PaCa-2 cells. Scale bar = 200 μm. Data are expressed as mean ± SD. All experiments were repeated three times independently. *P < 0.05, **P < 0.01, ***P < 0.001.

Given the close genomic proximity of NR2F1-AS1 and *NR2F1*, we hypothesized that NR2F1-AS1 could exert its effects by modulating *NR2F1*. *NR2F1* knockdown was achieved in PC cells using two siRNAs; knockdown was verified at the mRNA and protein levels (Supplementary Fig. S6A, B). As shown in a series of functional assays, silencing *NR2F1* resulted in significantly slower growth and fewer colonies (Fig. 5A–C). Wound-healing and transwell assays demonstrated that *NR2F1* knockdown reduced the migratory and invasive activities of PC cells (Fig. 5D–F). Therefore, the above results indicated that *NR2F1* might be a critical target of NR2F1-AS1 in its tumor-promoting role in PC.

### NR2F1-AS1/NR2F1 activates the AKT/mTOR pathway in PC cells

**Fig. 6:**
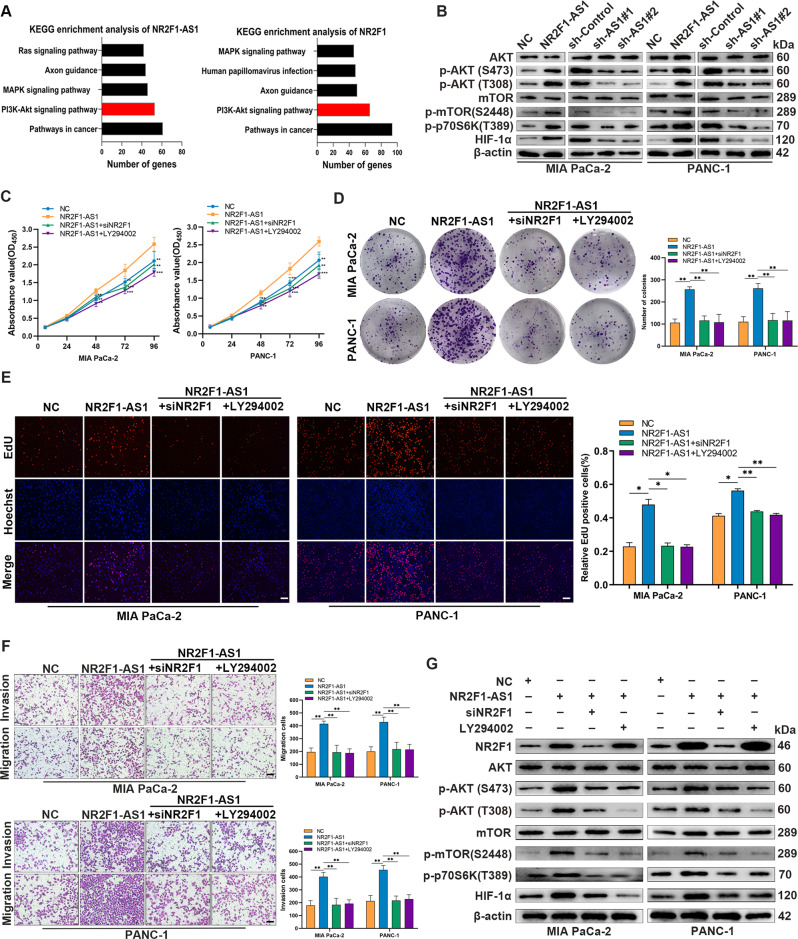
NR2F1-AS1/NR2F1 activates the AKT/mTOR pathway in PC cells. **A** The pathway analysis of NR2F1-AS1 with co-expressed genes using COEXPREdb (http://coxpresdb.jp/) by the KEGG. **B** Western blot analysis of AKT, p-AKT, mTOR, p-mTOR, p70 S6K, and HIF-1α in the indicated PC cells. **C** CCK-8 assays in the NC, NR2F1-AS1, NR2F1-AS1 + siNR2F1, and NR2F1-AS1 + LY294002 groups of PANC-1 and MIA PaCa-2 cells. **D** Colony-formation assays in the indicated PC cells. **E** EdU assays in the indicated PC cells. **F** Transwell assays showing the migration and invasion abilities of the indicated PC cells. **G** Western blot analysis of NR2F1, AKT, p-AKT, mTOR, p-mTOR, p70 S6K, and HIF-1α in the indicated PC cells. Scale bar = 200 μm. Data are expressed as mean ± SD. All experiments were repeated three times independently. *P < 0.05, **P < 0.01, ***P < 0.001.

To identify intracellular pathways that might mediate the effects of NR2F1 in PC cells, we performed bioinformatic analysis of similar genes from the TCGA-PAAD dataset. Kyoto Encyclopedia of Genes and Genomes (KEGG) pathway and correlation analysis showed that NR2F1-AS1 and NR2F1 were associated with the PI3K/AKT pathway (Fig. 6A and Supplementary Fig. S7A). Western blot analysis of MIA PaCa-2 and PANC-1 cells revealed that levels of the active, phosphorylated (p) forms of AKT, mTOR, and p70 S6K, and HIF-1α protein expression were obviously increased in PC cells overexpressing NR2F1-AS1 but were decreased in NR2F1-AS1-knockdown cells (Fig. 6B and Supplementary Fig. S7B, C). Thus, we hypothesized that NR2F1-AS1/NR2F1 might regulate PC progression by activating the PI3K/AKT pathway. Rescue experiments showed that both silencing NR2F1 and the PI3K inhibitor LY294002 blocked the NR2F1-AS1-mediated increased proliferation, migration, and invasion capacity of PC cells (Fig. 6C–F). Western blot analysis revealed that NR2F1, HIF-1α, p-AKT, p-mTOR, and p-p70 S6K levels were increased in NR2F1-AS1-overexpressing cells but were decreased in cells with NR2F1 knockdown (Fig. 6G and Supplementary Fig. S7D, E). Furthermore, our results indicated that LY294002 treatment could reverse the upregulation of p-AKT, p-mTOR, p-p70 S6K, and HIF-1α induced by NR2F1-AS1-overexpression but could not rescue the expression of NR2F1 (Fig. 6G and Supplementary Fig. S7D, E). Moreover, IHC analysis of 76 PC tissues indicated that NR2F1-AS1 expression was positively correlated with NR2F1 and HIF-1α (Supplementary Fig. S7F, G). These results indicated that NR2F1-AS1 promoted cell proliferation and invasion through NR2F1-mediated activation of AKT/mTOR signaling in PC cells.

### Hypoxia is positively correlated with NR2F1-AS1 expression

**Fig. 7:**
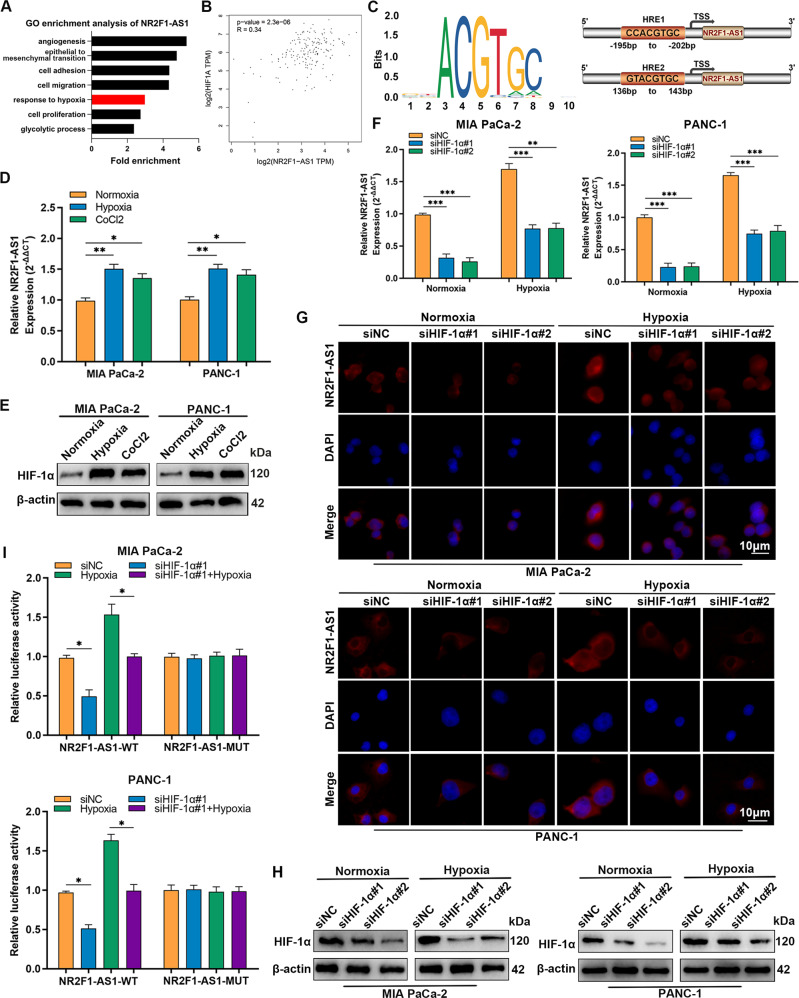
Hypoxia is positively correlated with NR2F1-AS1 expression. **A** GO enrichment analysis of NR2F1-AS1-related genes in TCGA-PAAD by the DAVID website. **B** NR2F1-AS1 expression was correlated with HIF-1α in the TCGA-PAAD data by GEPIA. **C** The recognition motif of HIF-1α from the JASPAR database and schematic illustration of two putative HREs (HRE1 and HRE2) in the NR2F1-AS1 promoter. **D**, **E** qRT-PCR and western blot results of NR2F1-AS1 and HIF-1α expression under normoxia, hypoxia (1% O_2_), or CoCl_2_ (100 μM). **F**–**H** qRT-PCR, western blot, and FISH assays were used to detect the expression of NR2F1-AS1 and HIF-1α under hypoxia or normoxia after silencing HIF-1α via siRNA. **I** MIA PaCa-2 and PANC-1 cells were transfected with siNC or siHIF-1α#1, and then further transfected with the pGL3 reporter vector containing the wild-type (WT) or mutated (MUT) NR2F1-AS1 HRE promoter under normoxia or hypoxia. Dual-luciferase report activity was detected and normalized. Scale bar = 10 μm. Data are expressed as mean ± SD. All experiments were repeated three times independently. *P < 0.05, **P < 0.01, ***P < 0.001.

To understand the regulatory mechanism that caused NR2F1-AS1 overexpression in PC, we used Gene Ontology (GO) enrichment analysis to discover that NR2F1-AS1 was responsive to hypoxia (Fig. 7A). Notably, HIF-1α expression, a critical regulator of hypoxia, was positively correlated with NR2F1-AS1 (Fig. 7B). Thus, we next analyzed the NR2F1-AS1 promoter using the JASPAR database, which identified two HREs in the NR2F1-AS1 promoter region (Fig. 7C and Supplementary Table S5). Subsequent results revealed that NR2F1-AS1 and HIF-1α expression were raised remarkably by incubating PC cells in a hypoxic microenvironment treatment for 24 h (Fig. 7D, E and Supplementary Fig. S8A). Next, we transfected PC cells with three different siRNAs targeting HIF-1α, and the interference efficiencies were verified by qRT-PCR and western blot (Supplementary Fig. S8B–D). Moreover, qRT-PCR and RNA-FISH results demonstrated that HIF-1α knockdown obviously inhibited NR2F1-AS1 expression in both normoxia and hypoxia conditions (Fig. 7F, G and Supplementary Fig. S8E). Western blot assays confirmed that HIF-1α expression was decreased in HIF-1α-silenced cells (Fig. 7H and Supplementary Fig. S8F, G). Luciferase reporter assays showed that hypoxia significantly enhanced the transcription of NR2F1-AS1-WT, which was repressed by HIF-1α knockdown (Fig. 7I). These data elucidated that NR2F1-AS1 is a hypoxia-responsive lncRNA in PC cells.

### NR2F1-AS1 participated in the hypoxia-induced metastasis of PC cells

**Fig. 8:**
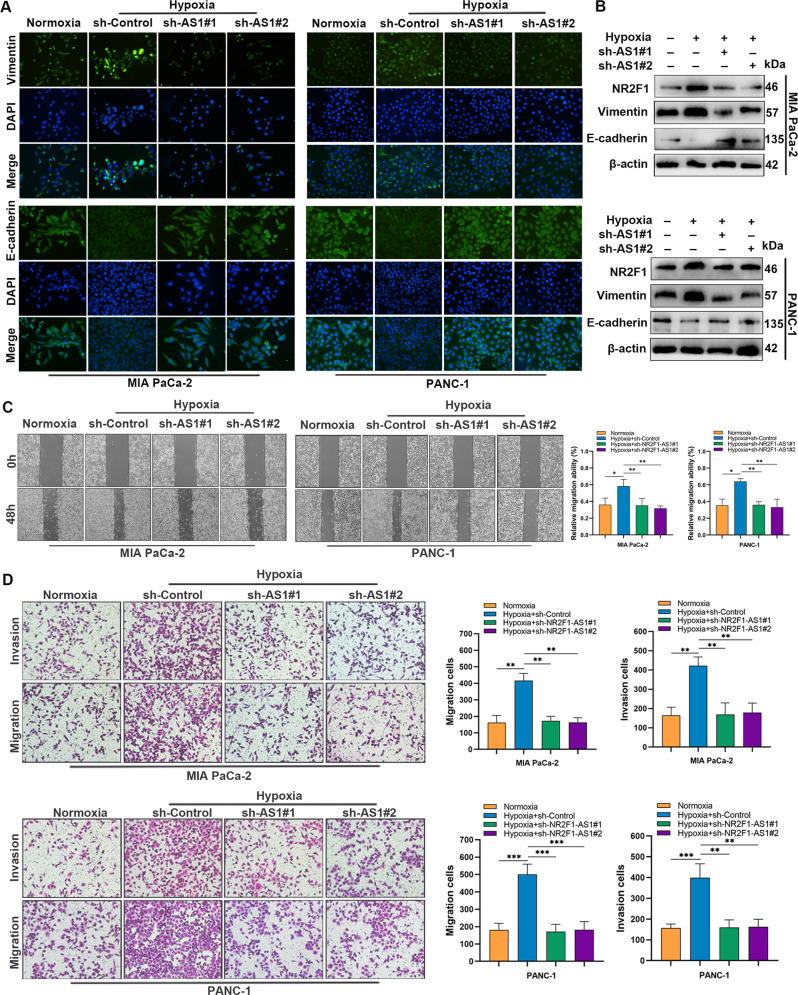
NR2F1-AS1 plays an important role in the hypoxia-induced migration and invasion of PC cells. **A** IF staining of epithelial-to-mesenchymal transition markers in the indicated cells; scale bar = 100 μm. **B** Western blot analysis of NR2F1, Vimentin, and E-cadherin expression in the indicated MIA PaCa-2 and PANC-1 cells. **C** Wound-healing assays were used to detect the migration ability of the indicated MIA PaCa-2 and PANC-1 cells. **D** Transwell assays were used to show the migration and invasion abilities of the indicated cells. For **C** and **D**, scale bar = 200 μm. Data are expressed as mean ± SD. All experiments were repeated three times independently. *P < 0.05, **P < 0.01, ***P < 0.001.

Next, we investigated the involvement of HIF-1α in the migration and invasion of PC cells by performing rescue experiments. The results revealed that NR2F1 and Vimentin expression were enhanced but E-cadherin was reduced under hypoxia, which was strikingly reversed by NR2F1-AS1 knockdown (Fig. 8A, B and Supplementary Fig. S9A, B). Similarly, the expression of NR2F1, Vimentin, and E-cadherin were restored in HIF-1α-silenced PC cells under hypoxia condition; overexpressing NR2F1-AS1 partially reversed the effect of HIF-1α knockdown in these cells (Supplementary Fig. S9C–E). We further explored the effect of hypoxia and HIF-1α on PC cell migration and invasion. Wound-healing and transwell assays demonstrated that hypoxia obviously enhanced the migration and invasion abilities of PC cells compared with cells cultured in normoxia; however, this effect was inhibited by knockdown of NR2F1-AS1 (Fig. 8C, D). Furthermore, silencing HIF-1α decreased the migration and invasion abilities induced by hypoxia, which were partially rescued by NR2F1-AS1 overexpression (Supplementary Fig. S10A, B). Therefore, the results all above implicate that NR2F1-AS1 participates in the hypoxia-induced metastasis of PC cells.

## Discussion

At present, many dysregulated lncRNAs have received extensive attention to elucidate their roles in the initiation and development of cancers [23]. Previously, NR2F1-AS1 was reported to play oncogenic roles in promoting the growth and metastasis of cancer [14–20]. Interestingly, Liu et al. found that NR2F1-AS1 is overexpressed in dormant mesenchymal-like breast cancer stem-like cells, where it functions to facilitate tumor dissemination but reduce proliferation, in a process that also involved NR2F1 [19]. In this study, we discovered that NR2F1-AS1 was highly expressed and positively correlated with larger tumor size, perineural invasion, and poor survival in PC. We further demonstrated that NR2F1-AS1 knockdown suppressed PC cell proliferation and metastasis in vitro and in vivo. Meanwhile, high NR2F1-AS1 expression facilitated hypoxia-induced EMT, migration, and invasion in PC cells. These data support our conclusion that NR2F1-AS1 may be an oncogene in PC, and thus it could be a potential prognostic biomarker for this disease.

Antisense lncRNAs have been reported to regulate the expression of their respective sense genes at multiple levels according to their subcellular localization [24]. For instance, the lncRNA EGFR-AS1 promotes the expression of EGFR by whittling the degradation of *EGFR* mRNA, thereby accelerating bladder cancer metastasis [25]. Wanowska et al. revealed that certain OIP5-AS1 splicing forms act as a scaffold for the SWI/SNF chromatin remodeling complex, which binds to the *OIP5* promoter, thus promoting its transcription [26]. Meanwhile, we found that NR2F1-AS1 is an antisense transcript of *NR2F1* that is located in the opposite strand of DNA, meaning NR2F1-AS1 could cis-regulate its neighbor *NR2F1*. Notably, we observed that NR2F1 was positively modulated by NR2F1-AS1 at both the mRNA and protein levels. We further explored the mechanism by which NR2F1-AS1 regulated NR2F1 expression and verified that NR2F1 is a pivotal target of NR2F1-AS1 in PC but understanding the underlying molecular mechanism will require further investigation.

Previous studies have confirmed that NR2F1 is a transcription factor [27], and it has been found to be an oncoprotein in various tumors [28]. In our functional assays, *NR2F1* depletion repressed the proliferation, migration, invasion, and rescued the phenotypes mediated by NR2F1-AS1. Based on KEGG pathway analysis, we found that both NR2F1-AS1 and NR2F1 were significantly related to the PI3K/AKT pathway, which controls key cellular processes such as metabolism, growth, invasion, and the survival, expansion, and spread of cancer cells [29]. Previous studies have demonstrated that lncRNA NR2F1-AS1 can influence breast and gastric cancer progression by activating insulin-like growth factor-1 (IGF-1)/IGF-1R/ERK [20], MAP3K2 [30], and AKT3 [31], which crosstalk with AKT. We also verified that NR2F1-AS1 promoted AKT/mTOR signaling, indicating that NR2F1-AS1 is a critical gene for the growth and metastasis of PC. Then, various functional rescue assays confirmed that AKT/mTOR signaling controlled key components through which NR2F1-AS1/NR2F1 drove PC progression. On the basis of these data, we concluded that NR2F1-AS1 continuously triggered activation of AKT/mTOR signaling by promoting NR2F1 expression. Interestingly, previous studies have confirmed that AKT/mTOR signaling stimulates *HIF-1α* mRNA expression and enhances HIF-1α protein stability [32, 33]. Therefore, we further explored the effect of NR2F1-AS1 on HIF-1α protein levels. As expected, silencing NR2F1-AS1 inhibited HIF-1α protein expression, while overexpressing NR2F1-AS1 promoted HIF-1α expression. In addition, the expression of HIF-1α was partially diminished by *NR2F1* knockdown and LY294002 treatment in NR2F1-AS1-overexpressing cells. Taken together, these data suggest a novel mechanism through which NR2F1-AS1 facilitates PC progression by promoting the expression of its neighbor *NR2F1*, which then activates the AKT/mTOR/HIF-1α axis.

The tumor microenvironment is an important feature of solid tumors that can activate the expression of specific genes to regulate various biological behaviors [34, 35]. Numerous studies have revealed that lncRNAs are upregulated by a hypoxic microenvironment, thereby promoting the occurrence and development of cancers, including PC [12, 36, 37]. According to one study, lincRNA-p21 is a hypoxia-responsive lncRNA that conversely regulates the stability of HIF-1α protein [38]. As our bioinformatics analysis predicted two potential HREs in the NR2F1-AS1 promoter, we further investigated whether NR2F1-AS1 was a hypoxia-sensitive lncRNA in PC cells. Our results showed that the expression and transcriptional activity of NR2F1-AS1 were elevated under hypoxia, owing to HIF-1α binding to the NR2F1-AS1 promoter. These data confirmed that NR2F1-AS1 regulates HIF-1α expression by modulating the activity of the AKT/mTOR pathway in PC. These findings are consistent with the concept of HIF-1α transcriptional activation of target lncRNAs and suggest a reciprocal feedback mechanism between NR2F1-AS1 and HIF-1α in PC cells under hypoxia.

**Fig. 9:**
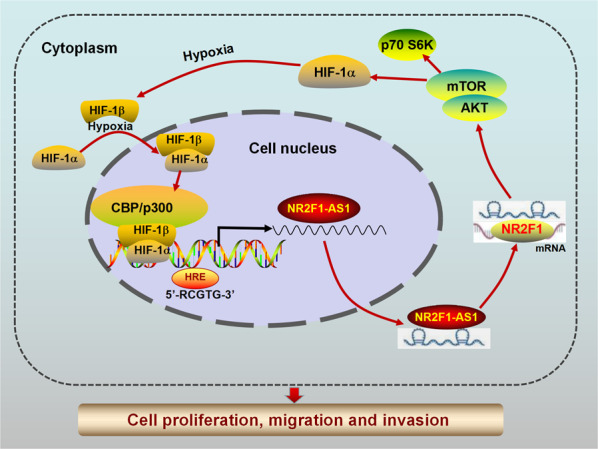
Schematic illustration of the functions of NR2F1-AS1 in pancreatic cancer. During pancreatic tumorigenesis, NR2F1-AS1 was transcriptionally upregulated by a hypoxia-inducible factor, HIF-1α. NR2F1-AS1 promoted PC cell proliferation, migration, and invasion via activating the NR2F1/AKT/mTOR pathway. Mechanistically, NR2F1-AS1 positively regulated the neighboring NR2F1 gene, which subsequently activated AKT/mTOR signaling, resulting in the upregulation of HIF-1α.

In summary, this study demonstrated that NR2F1-AS1 is a novel hypoxia-inducible lncRNA that is highly expressed and related to poor prognosis in PC patients. NR2F1-AS1 is regulated by HIF-1α and promotes PC cell proliferation, migration, and invasion by maintaining the expression of its sense gene *NR2F1*, which activates the AKT/mTOR pathway (Fig. 9). Our data describe a novel mechanism for PC progression, which might be beneficial for discovering therapeutic targets.

## Supplementary information

Supplementary materials and methods

Supplementary figure and table legends

Supplementary Figures

Supplementary Tables

Supplementary Table S5

Reproducibility checklist

IRB approval

The original data of WB blots

## Data Availability

The datasets used and analyzed during this study are available from the corresponding author on reasonable request.
