# Supplementary material for: Hypoxia-induced long noncoding RNA NR2F1-AS1 maintains pancreatic cancer proliferation, migration, and invasion by activating the NR2F1/AKT/mTOR axis
Source: Cell Death Dis. 2022 Mar 14;13(3):232. doi: 10.1038/s41419-022-04669-0 (PMC8918554; doi:10.1038/s41419-022-04669-0)
Supplement: Supplementary file 1 — Supplementary materials and methods [file 41419_2022_4669_MOESM1_ESM.doc]

**Supplementary materials and methods**

**RNA extraction and quantitative real-time polymerase chain reaction (****qRT-PCR)**

Total RNA was extracted from tissues and cells using Trizol kits (Invitrogen, Carlsbad, CA, USA), and the concentration and optical density were assessed by a spectrophotometer. The absorbance ratio at 260 nm/280 nm ranging from 1.8 to 2.0 indicated purity of extracted RNA. RNA samples for mRNA and lncRNA were reversely transcribed into cDNA using the PrimeScript™ RT reagent Kit (TaKaRa Biotechnology Co. Ltd., Liaoning China). PCR was conducted using the TB Green® Premix Ex Taq™ II PCR Kit (TaKaRa Biotechnology Co. Ltd., Liaoning China) on the CFX96 Real-Time PCR Detection System (CFX96; Bio-Rad, Hercules, CA, USA). The primers were designed and synthesized by Sangon Biotech Co., Ltd. (Shanghai, China) and Guangzhou RiboBio Co., Ltd. (Guangzhou, China), and the sequences were listed in Table S1. U6 and beta-actin (β-actin) mRNA were used as internal references. The data were analyzed by the 2^-ΔΔCT^ method.

**Western blot analysis**

Cells were washed with pre-cooled PBS three times and lysed using RIPA lysis buffer (Solarbio, [Beijing](../../../../D:/%25E5%25AE%259E%25E9%25AA%258C%25E6%2595%25B0%25E6%258D%25AE%25E5%25A4%2584%25E7%2590%2586%25E8%25BD%25AF%25E4%25BB%25B6/Dict/8.9.6.0/resultui/html/index.html#/javascript:;), China) for 30 min. Samples were centrifuged at 12,000 rpm, and the protein concentration of the supernatant was determined using the BCA protein assay kit (Beyotime, Shanghai, China). Lysates were mixed with 5×loading buffer, boiled at 95°C for 5 min, and separated by electrophoresis and transferred onto PVDF membranes (Milipore Co. USA). The membranes were incubated with primary antibodies at 4 °C for 16 h-18h. The membranes were then blocked for 1 h in 5% TBST milk and then incubated with specific HRP-conjugated secondary antibody at room temperature for 2 h. Bands were visualized using ECL reagent (Milipore Co. USA) and the Bio-Rad Imaging System. The primary and second antibodies are shown in Table S3.

**Subcellular fractionation analysis**

Subcellular isolation of RNAs in MIA PaCa-2 and PANC-1 cells was conducted by Cytoplasmic and Nuclear RNA Purification Kit (Norgenbiotek Corporation, Thorold, ON, Canada) according to the manufacturer’s instructions. Cytoplasmic and nuclear fractions were determined by qRT-PCR. β-actin and U6 RNA levels in cytoplasm and nuclear fraction act as the control, respectively.

**Cell viability assay**

Cells (2×10^3^/well) were seeded in 96-well plates and transfected with sh-NR2F1-AS or siNR2F1 or NR2F1-AS1+siNR2F1/LY29004 (Cell Signal Technology,USA) for 6, 24, 48, 72 or 96 h. Cell counting kit-8 (CCK-8) reagent (Dojindo Laboratories, Kumamoto, Japan) was added to each well (10 μL), and cells were incubated for 2 h. The optical densities at 450 nm were measured using a microplate reader (Molecular Devices Sunnyvale, CA, USA).

**Colony formation assay**

Stable transfected cells in the logarithmic growth phase were detached by trypsin and plated in 6-well plates at 500 cells/well. Three duplicate wells were set in each group. After being cultured for 14 d, the cells were washed by phosphate buffered saline (PBS) three times, fixed in 4% paraformaldehyde and stained with 0.1% crystal violet staining solution for 30 min. Stained colonies were counted under an inverted microscope.

**Wound healing assay**

Transfected or treated with different ways cells were plated in 6-well plates and allowed to grow to confluence. The cell monolayer was scratched a 200 μL pipette tip to create a wound scratch. The indicated cells were deprived of serum for 48 h and treated with mitomycin-C. Photos were captured at 0 and 48 h after scraping.

**Transwell assays**

Transwell chambers (Corning Costar, NY, USA) were used for migration experiments, and chambers coated with 1:8 diluted Matrigel (BD Biosciences, Franklin Lakes, NY, USA) were used for invasion experiments, respectively. Transfected cells in the logarithmic growth phase were collected and treated with serum-free medium for 8-12 h. The cells were detached by trypsin, and 5×10^4^ cells were suspended in the upper chambers with 200 μL serum-free medium, then 600 μL medium containing 20% serum was placed in the lower chambers and incubated at 37°C for 24 h. The cells were fixed in 4% paraformaldehyde for 30 min, stained by 0.1% crystal violet staining solution for 15 min and photographed under a microscope, then counted in 5 random fields of view.

**5-Ethynyl-2′-deoxyuridine (EdU) labeling assay**

EdU kits were purchased from Guangzhou RiboBio Co., Ltd. Transfected cells in the logarithmic growth phase were collected and seeded into 24-well plates at 4×10^4^ cells/well for 24 h. When the cell confluence reached 60%, 300 μL 50μM diluted EdU solution was added to each well and plates and then incubated for 2 h. The cells were fixed and stained based on the instructions of the EdU kit. Cells were observed and photographed under a fluorescence microscope, and cells in four random fields of view were counted to calculate the proliferation rate.
