# Supplementary material for: Hypoxia-induced long noncoding RNA NR2F1-AS1 maintains pancreatic cancer proliferation, migration, and invasion by activating the NR2F1/AKT/mTOR axis
Source: Cell Death Dis. 2022 Mar 14;13(3):232. doi: 10.1038/s41419-022-04669-0 (PMC8918554; doi:10.1038/s41419-022-04669-0)
Supplement: Supplementary file 2 — Supplementary figure and table legends [file 41419_2022_4669_MOESM2_ESM.doc]

**Supplementary Tables:**

**Supplementary Table S1. The primer sequences of qRT-PCR.**

**Supplementary Table S2. Sequences of siRNAs/shRNAs used in this study.**

**Supplementary Table S3. Antibodies used in this study.**

**Supplementary Table S4. Correlation between NR2F1-AS1 expression and the clinicopathological features of pancreatic cancer.**

**Supplementary Table S5. Results of predicted transcription factors that can bind to NR2F1-AS1.**

[**Supplementary Figure legend**](https://static-content.springer.com/esm/art:10.1038/s41419-020-2569-y/MediaObjects/41419_2020_2569_MOESM1_ESM.docx)**s:**

**Supplementary Figure S1. Expression of NR2F1-AS1 is upregulated in PC tumor tissues and metastatic tissues from the GSE datasets, and is not related to clinicopathological characteristics from the TCGA data.**

**Supplementary Figure S2. NR2F1-AS1 is an oncogenic gene in PC cells.**

**Supplementary Figure S3. NR2F1-AS1 facilitates the proliferation, migration and invasion of PC cells.**

**Supplementary Figure S4. NR2F1-AS1 knockdown attenuates tumor growth and metastasis in nude mice.**

**Supplementary Figure S5. NR2F1 is a key target of NR2F1-AS1 in PC cells.**

**Supplementary Figure S6. The NR2F1 expression level in PC cells after transfected with siNR2F1s.**

**Supplementary Figure S7. The correlation of NR2F1-AS1, NR2F1 and AKT in TCGA-PAAD data, PC cells and tissues.**

**Supplementary Figure S8. HIF-1α regulates positively NR2F1-AS1 expression in PC cells.**

**Supplementary Figure S9. Effect of NR2F1-AS1 on NR2F1, Vimentin, E-cadherin in hypoxia microenvironment of PC cells.**

**Supplementary Figure S10. Effect of NR2F1-AS1 on hypoxia-induced migration and invasion in PC cells.**
