# Supplementary material for: Hypoxia-induced long noncoding RNA NR2F1-AS1 maintains pancreatic cancer proliferation, migration, and invasion by activating the NR2F1/AKT/mTOR axis
Source: Cell Death Dis. 2022 Mar 14;13(3):232. doi: 10.1038/s41419-022-04669-0 (PMC8918554; doi:10.1038/s41419-022-04669-0)
Supplement: Supplementary file 3 — Supplementary Figures [file 41419_2022_4669_MOESM3_ESM.doc]

**Supplementary Figures**

**Supplementary Figure S1**


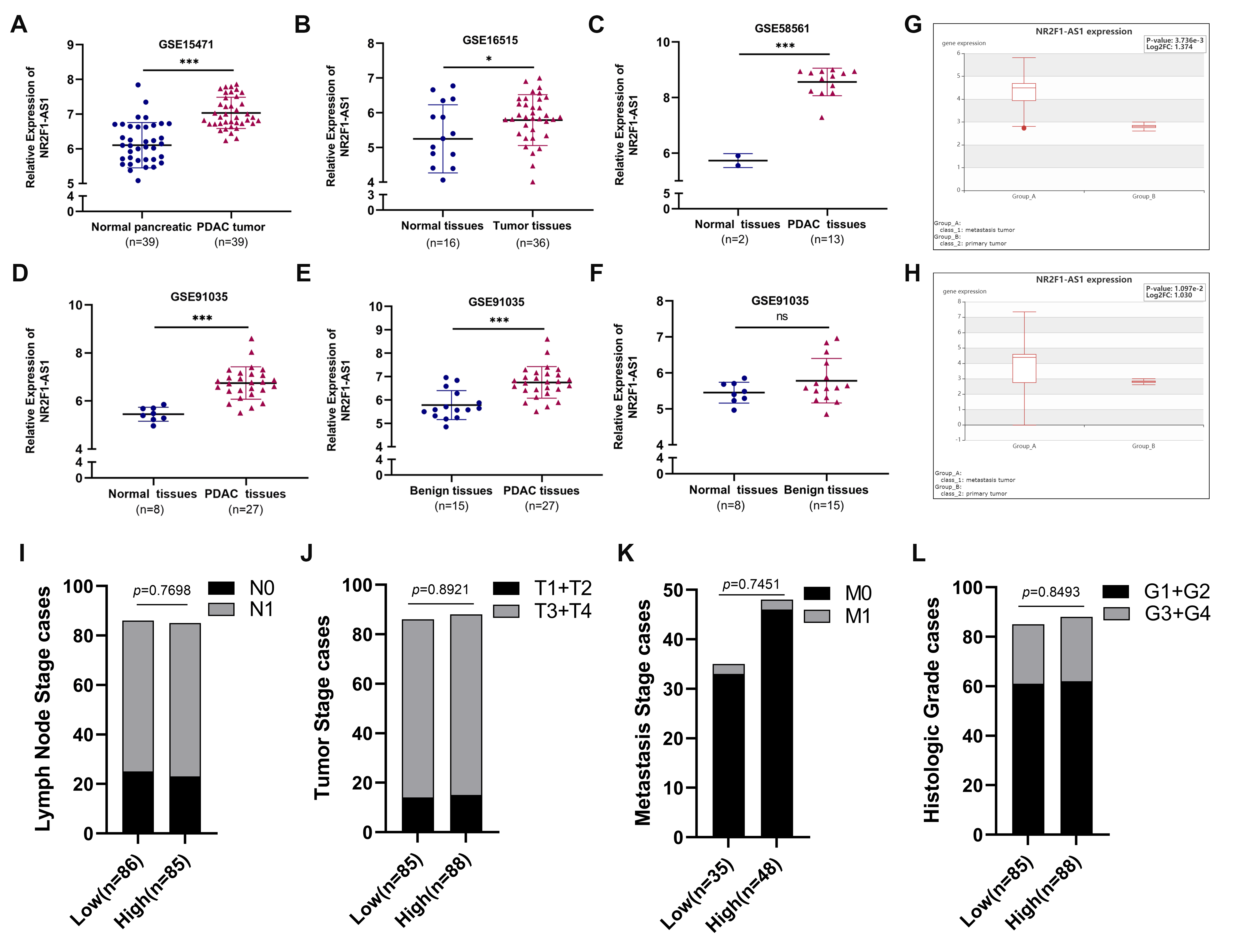


**Supplementary Figure S1. Expression of NR2F1-AS1 is upregulated in PC tumor tissues and metastatic tissues from the GSE datasets, and is not related to** **clinicopathological characteristics from the TCGA data.**

**A** Whole-Tissue Gene Expression Study of Pancreatic Ductal Adenocarcinoma (PDAC) with GSE15471 showed that NR2F1-AS1 was highly expressed in PDAC. **B** GSE16515 about expression data from Mayo Clinic Pancreatic Tumor and Normal samples showed that NR2F1-AS1 was highly expressed in pancreatic tumors. **C** GSE58561 contained Gene expression profiles for PDAC cell lines and their corresponding normal and fresh frozen tissues showed that NR2F1-AS1 was highly expressed in PDAC. **D-F** The gene expression signatures of human normal, benign and PDAC pancreatic tissues in GSE91035 showed high expression of NR2F1-AS1 in PDAC (**D**) and low expression in normal (**E**) and benign (**F**) pancreatic tissues. **G** The expression of NR2F1-AS1 in lung metastasis tumor is higher than PC primary tumor from GSE63124 by HCMDB (https://hcmdb.i-sanger.com/). **H** The expression of NR2F1-AS1 in lung, liver and peritoneum metastasis tumor is higher than PC primary tumor from GSE63124 by HCMDB. **I-L** Graphical illustration of NR2F1-AS1 expression distribution in TCGA data for N stages (I), T stages (J), M stages (K), and histologic grades (L). **P*<0.05, ***P*<0.01, ****P*<0.001

**Supplementary Figure S2**


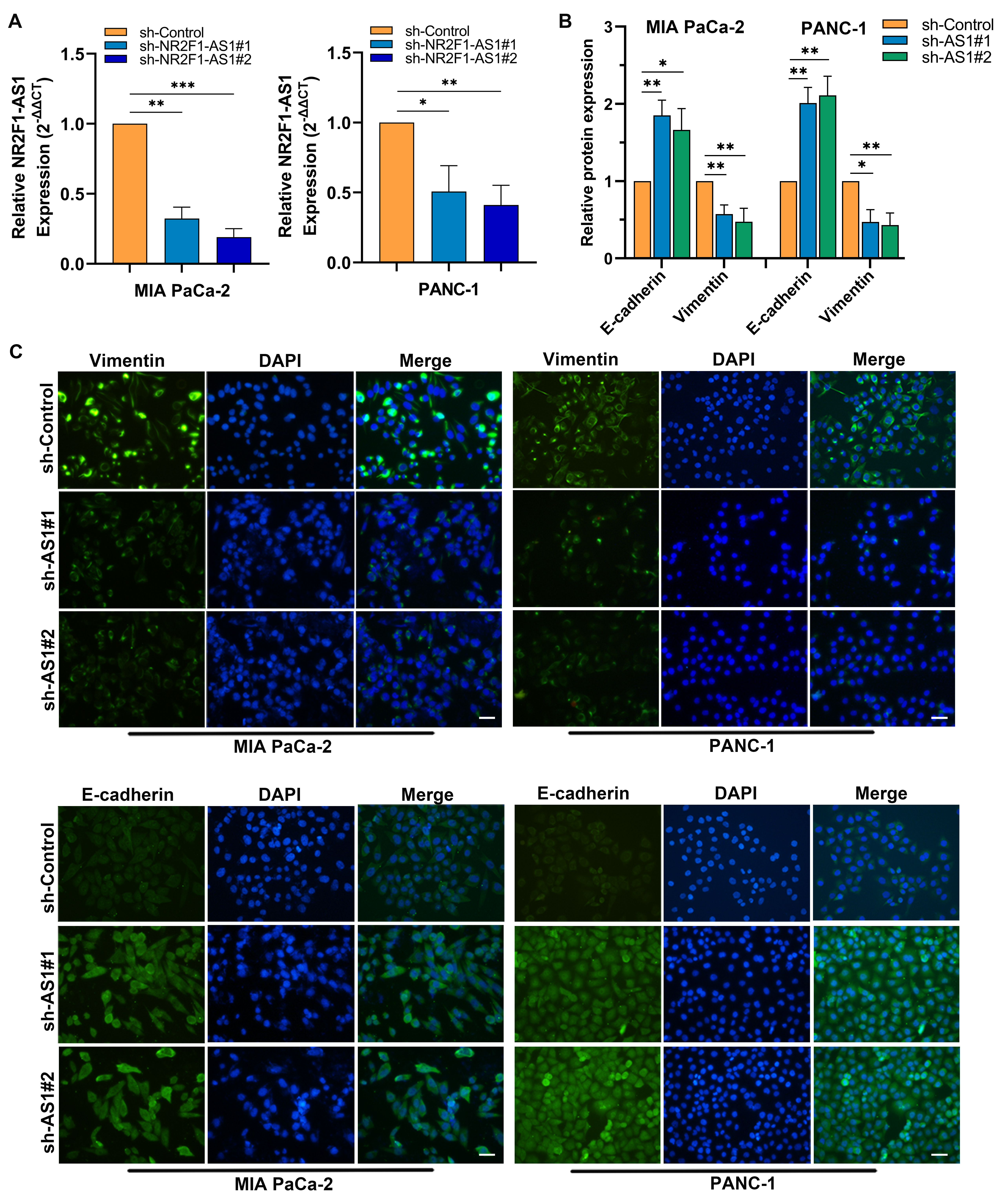


**Supplementary Figure S2. NR2F1-AS1 is an oncogenic gene in PC cells.**

**A** qRT-PCR analysis showed NR2F1-AS1 expression after transfection of NR2F1-AS1-knockdown constructs (sh-NR2F1-AS1#1 and sh-NR2F1-AS1#2) or the negative control (sh-Control) in PANC-1 and MIA PaCa-2 cells using lentiviral infection. **B** Illustration of E-cadherin and Vimentin protein quantification in different treatment groups were confirmed by western blot in **Fig. 2F. C** IF staining analysis of Vimentin and E-cadherin expression in NR2F1-AS1-knockdown and sh-Control groups in MIA PaCa-2 and PANC-1 cells. Scale bar = 100μm. Data were expressed as mean ± SD. All experiments were repeated three times independently. **P*<0.05, ***P*<0.01, ****P*<0.001.

**Supplementary Figure S3**


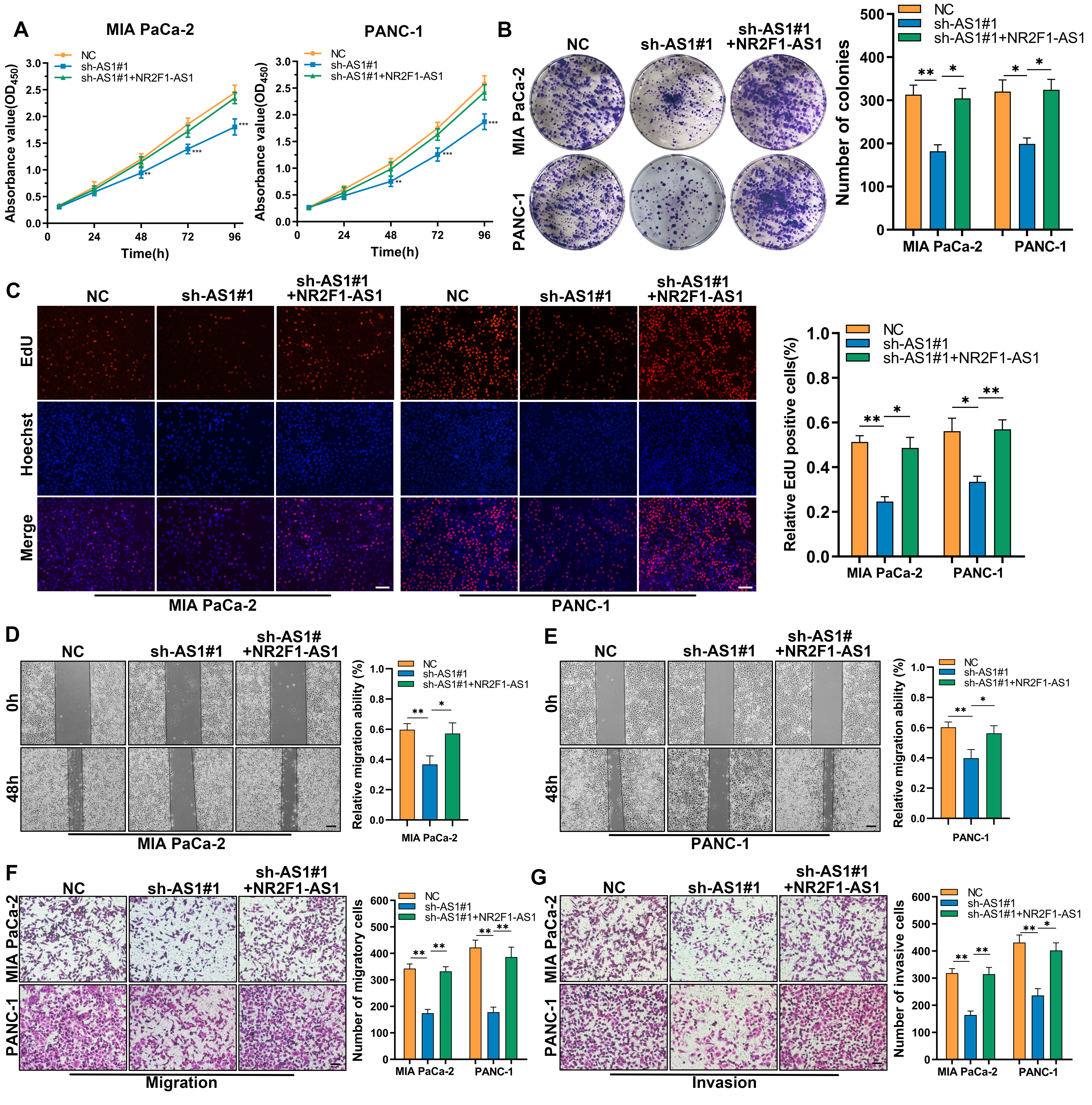


**Supplementary Figure S3. NR2F1-AS1 facilitates the proliferation, migration and invasion of PC cells. A-C** Cell proliferation was performed by CCK-8, colony formation, and EdU staining assays in MIA PaCa-2 and PANC-1 cells transfected with negative control (NC), sh-NR2F1-AS1#1 and rescue (sh-NR2F1-AS1#1+NR2F1-AS1) groups. **D-G** Cell migration and invasion ability were examined by wound healing and transwell assays in MIA PaCa-2 and PANC-1 cells transfected with negative control (NC), sh-NR2F1-AS1#1 and rescue (sh-NR2F1-AS1#1+NR2F1-AS1) groups. Scale bar = 200μm. Data were expressed as mean ± SD. All experiments were repeated three times independently. **P*<0.05, ***P*<0.01, ****P*<0.001.

**Supplementary Figure S4**


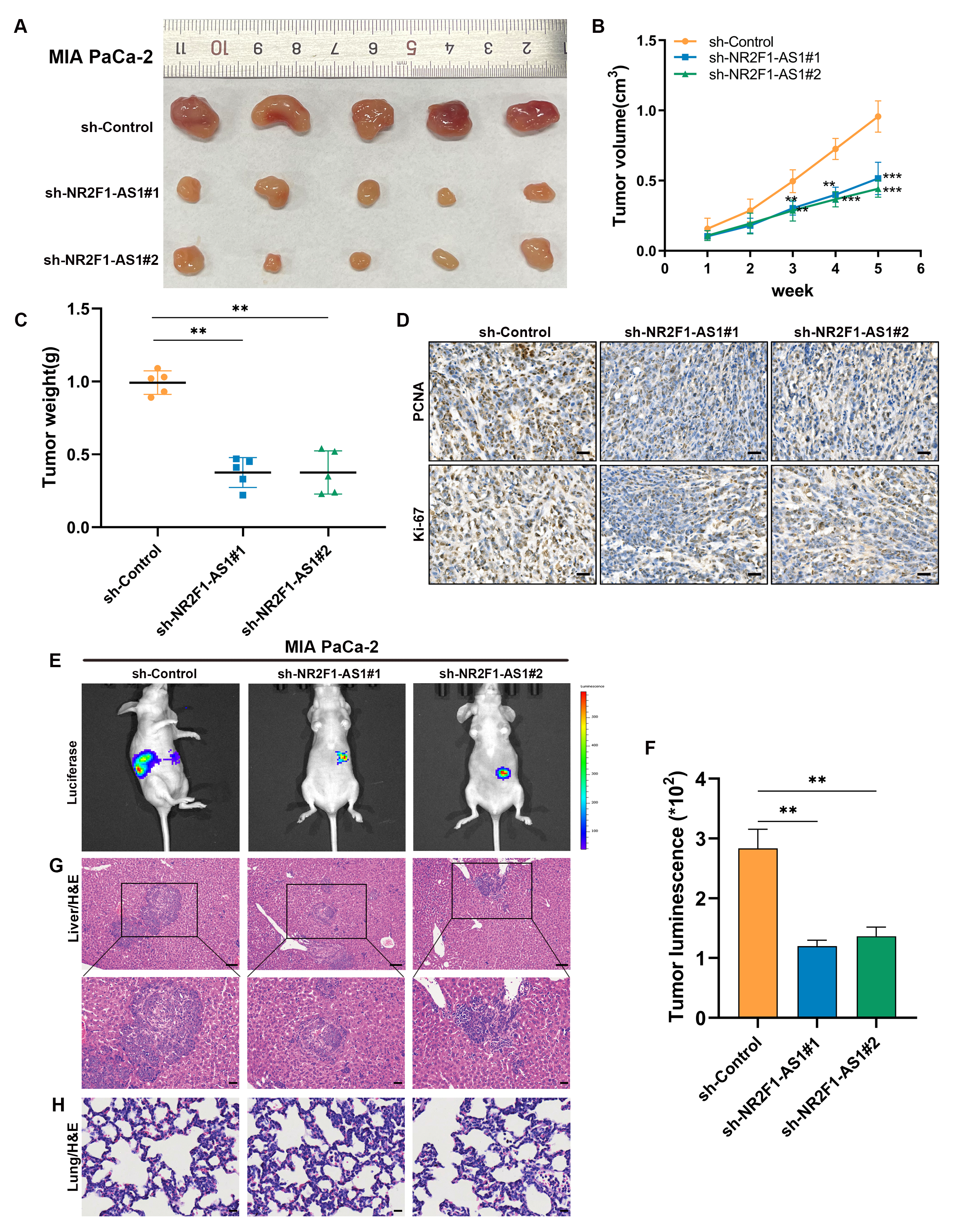


**Supplementary Figure S4. NR2F1-AS1 knockdown attenuates tumor growth and metastasis in nude mice. A** Image of xenograft tumors resected from nude mice-carrying MIA PaCa-2 cells transfected using lentiviruses encoding sh-Control, sh-NR2F1-AS1#1 or sh-NR2F1-AS1#2, (n=5). **B** The volume of subcutaneous tumor was measured every week. **C** The weight of subcutaneous tumor was measured after 5 weeks while mice were sacrificed in each group. **D** Representative images of IHC staining of Ki-67 and PCNA protein expression levels in the tumor sections from sh-Control, sh-NR2F1-AS1#1 or sh-NR2F1-AS1#2, Scale bar = 100μm. **E-H** Representative fluorescent images of microscopic metastatic nodules generation were performed after 5 weeks and the liver (**G**) or lung (**H**) tissues stained with haematoxylin and eosin (H&E), Scale bar = 20μm. Data were expressed as mean ± SD. **P*<0.05, ***P*<0.01, ****P*<0.001.

**Supplementary Figure S5**


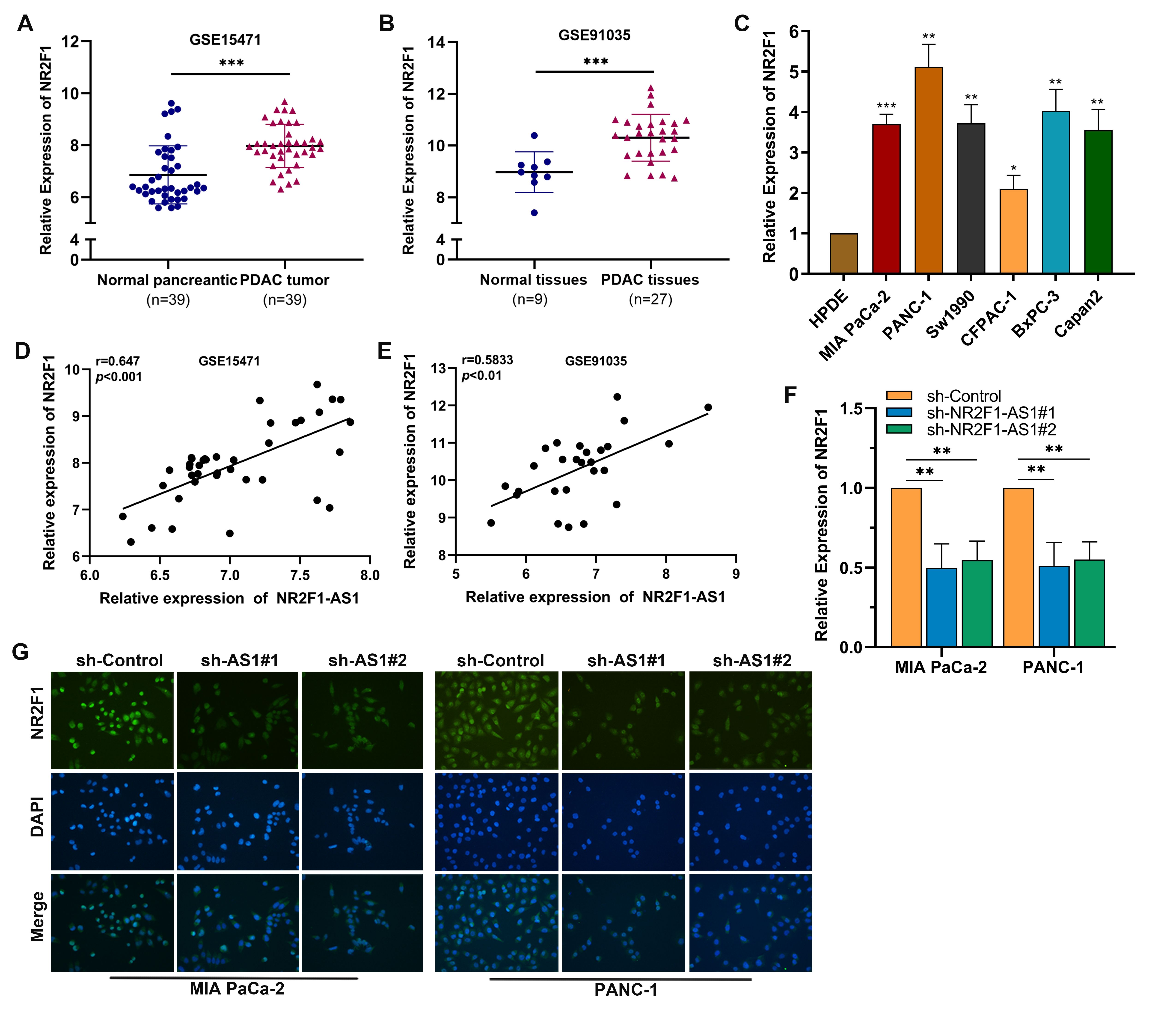


**Supplementary Figure S5. NR2F1 is a key target of NR2F1-AS1 in PC cells.**

**A** GSE15471 showed that NR2F1 was highly expressed in PDAC. **B** NR2F1 expression levels was upregulated in PDAC tissues of the GSE91035 microarray. **C** Illustration of NR2F1 protein quantification in PC and HPDE cell lines were confirmed by western blot in **Fig. 4F. D** The expression of NR2F1 showed positive correlation with NR2F1-AS1 expression in GSE15471. **E** A positive correlation was demonstrated between NR2F1-AS1 and NR2F1 expression by Pearson’s correlation analysis in GSE91035. **F,G** The expression of NR2F1 was downregulated in NR2F1-AS1-knockdown compared with control groups in MIA PaCa-2 and PANC-1 cells by western blot in **Fig. 4J** (**F**) and IF staining (**G**) analysis. Scale bar = 100μm. Data were expressed as mean ± SD. Cell experiments were repeated three times independently. **P*<0.05, ***P*<0.01, ****P*<0.001.

**Supplementary Figure S6**


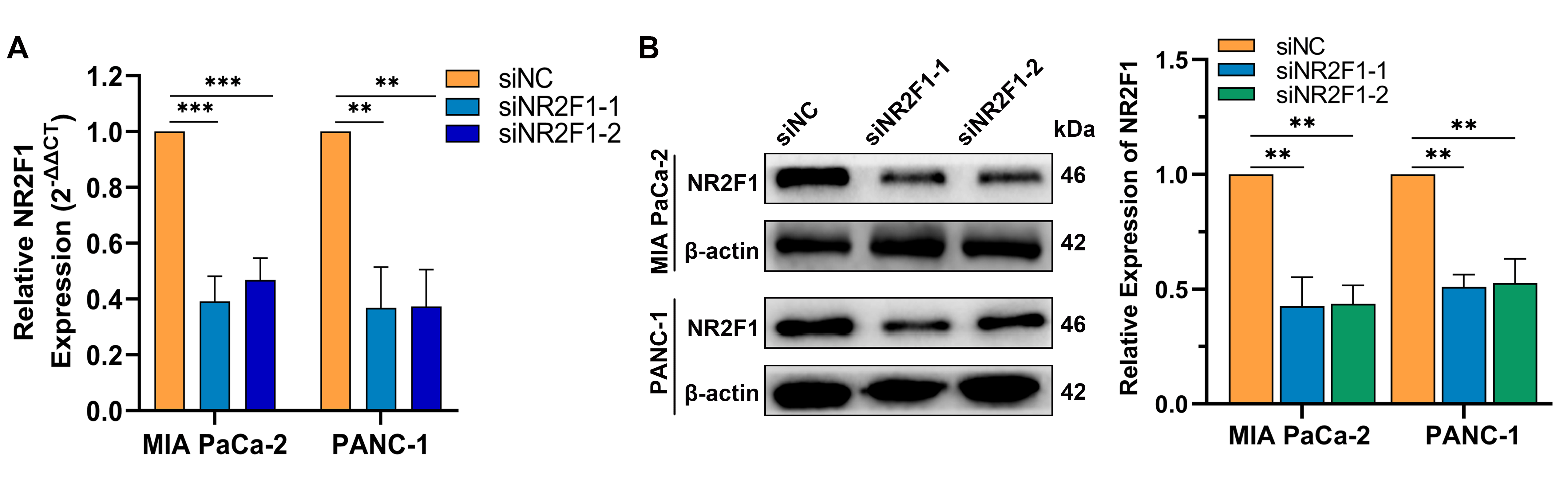


**Supplementary Figure S6. The NR2F1 expression level in PC cells after transfected with siNR2F1s.**

qRT-PCR (**A**) and western blot (**B**) analysis of NR2F1 was downregulated after being transfected using siNR2F1-1 and siNR2F1-2 in MIA PaCa-2 and PANC-1 cells. Data were expressed as mean ± SD. All experiments were repeated three times independently. ***P*<0.01, ****P*<0.001.

**Supplementary Figure S7**


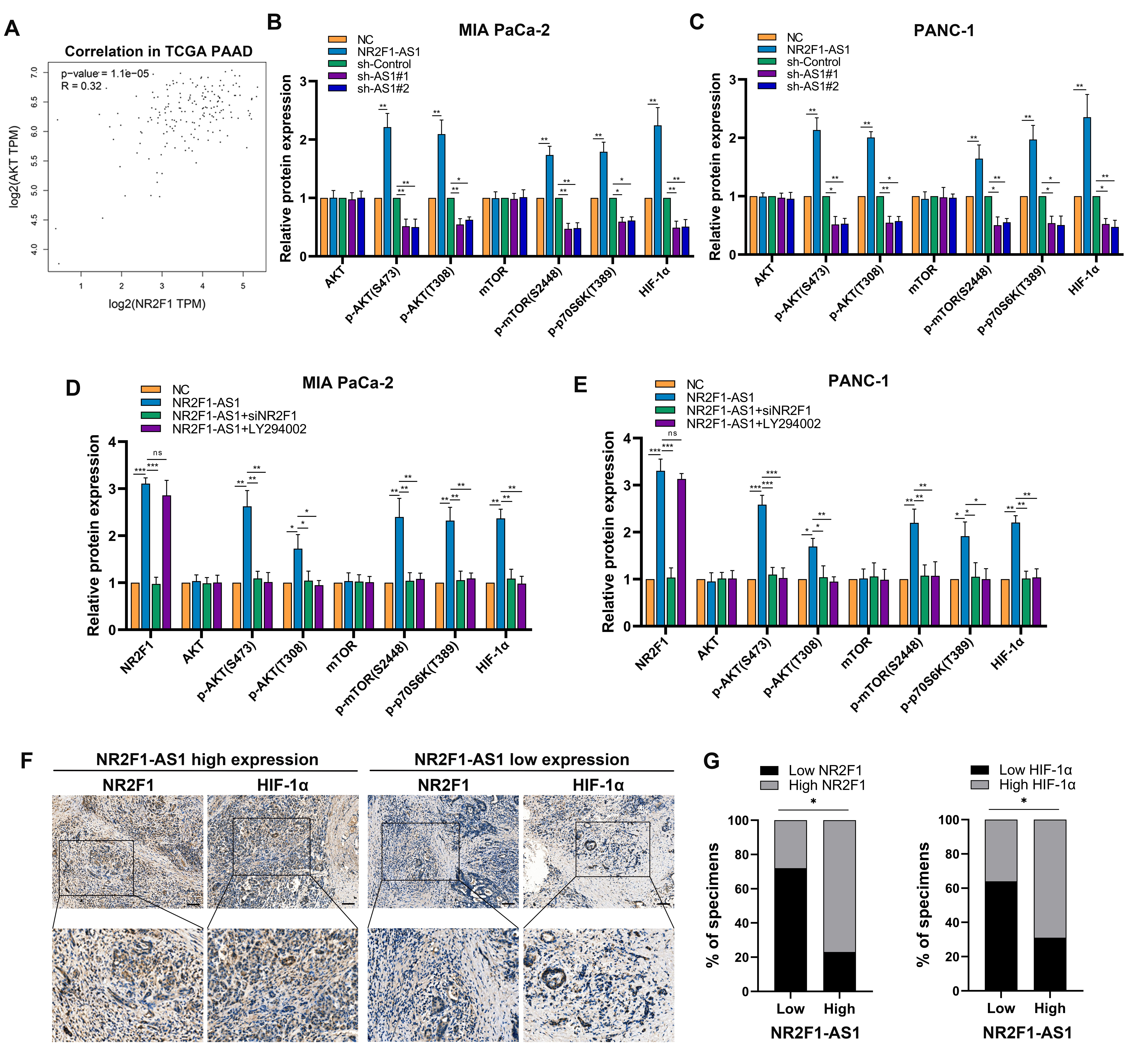


**Supplementary Figure S7. The correlation of NR2F1-AS1, NR2F1 and AKT in TCGA-PAAD data, PC cells and tissues.**

**A** NR2F1 expression is positively correlated with AKT1 expression in TCGA-PAAD data on GEPIA website. **B,C** Illustration of relative proteins quantification in different treatment groups were detected by western blot in **Fig. 6B. D,E** Illustration of relative protein quantification in different treatment groups were detected by western blot in **Fig. 6G. F** IHC analysis of NR2F1 and HIF-1α expression in serial sections of PC specimens, scare bar = 200 μm. **G** The expression level of NR2F1-AS1 correlates with NR2F1 and HIF-1α in PC specimens. Data were expressed as mean ± SD. Cell experiments were repeated three times independently. **P*<0.05, ***P*<0.01, ****P*<0.001.

**Supplementary Figure S8**


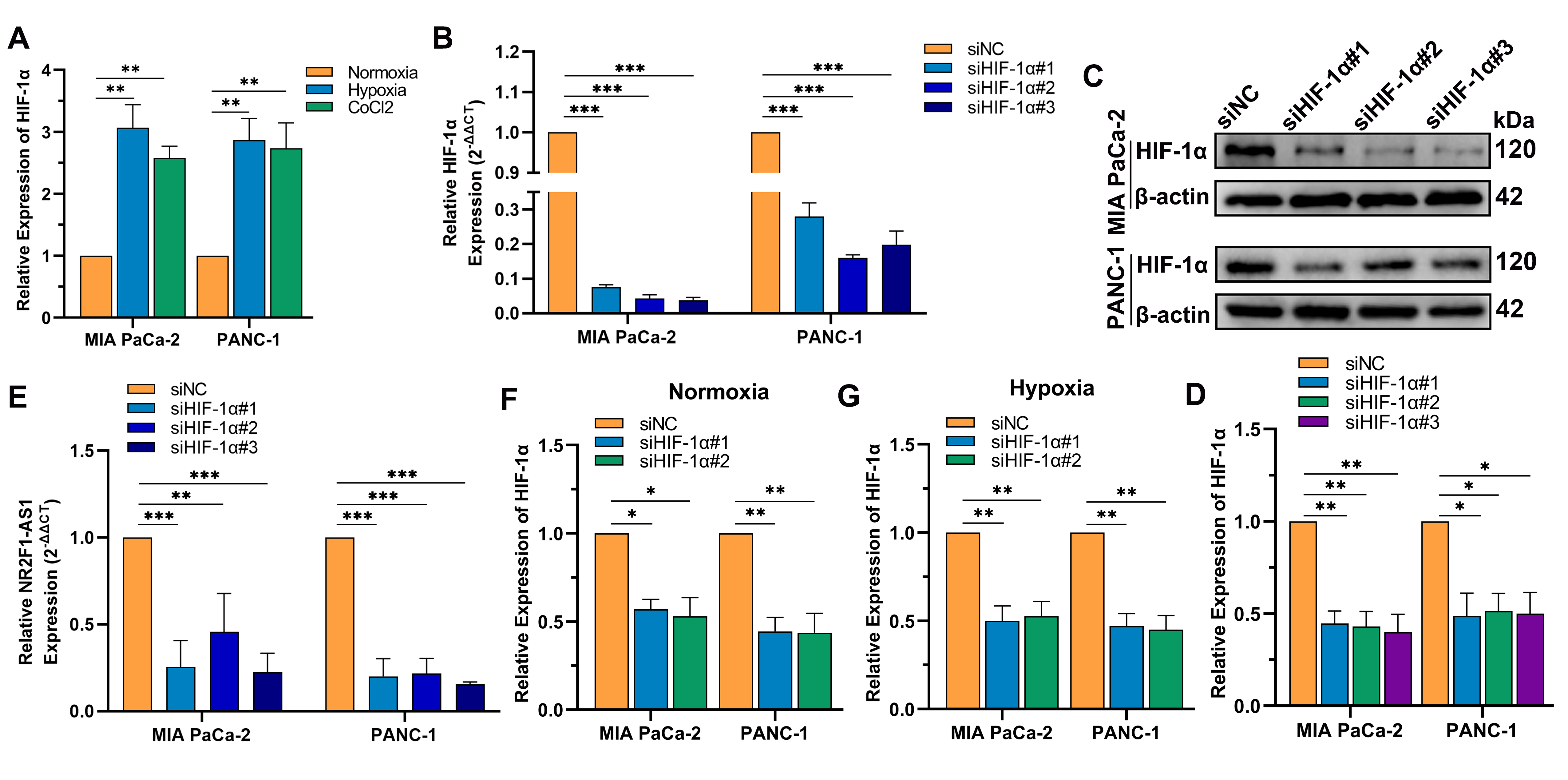


**Supplementary Figure S8. HIF-1α regulates positively NR2F1-AS1 expression in PC cells.**

**A** Illustration of HIF-1α protein quantification in different treatment groups were detected by western blot in **Fig. 7E. B-D** The mRNA and protein expression levels of HIF-1α was knocked down by transfection with three siRNAs targeting HIF-1α which detected by qRT-PCR (**B**) and western blot (**C,D**) analysis in PC cells. **E** qRT-PCR results showed that down-regulation of HIF-1α could effectively inhibit NR2F1-AS1 mRNA expression in MIA PaCa-2 and PANC-1 cells. **F,G** Illustration of HIF-1α protein quantification in different treatment groups were performed by western blot in **Fig. 7H.** Data were expressed as mean ± SD. All experiments were repeated three times independently. **P*<0.05, ***P*<0.01, ****P*<0.001.

**Supplementary Figure S9**


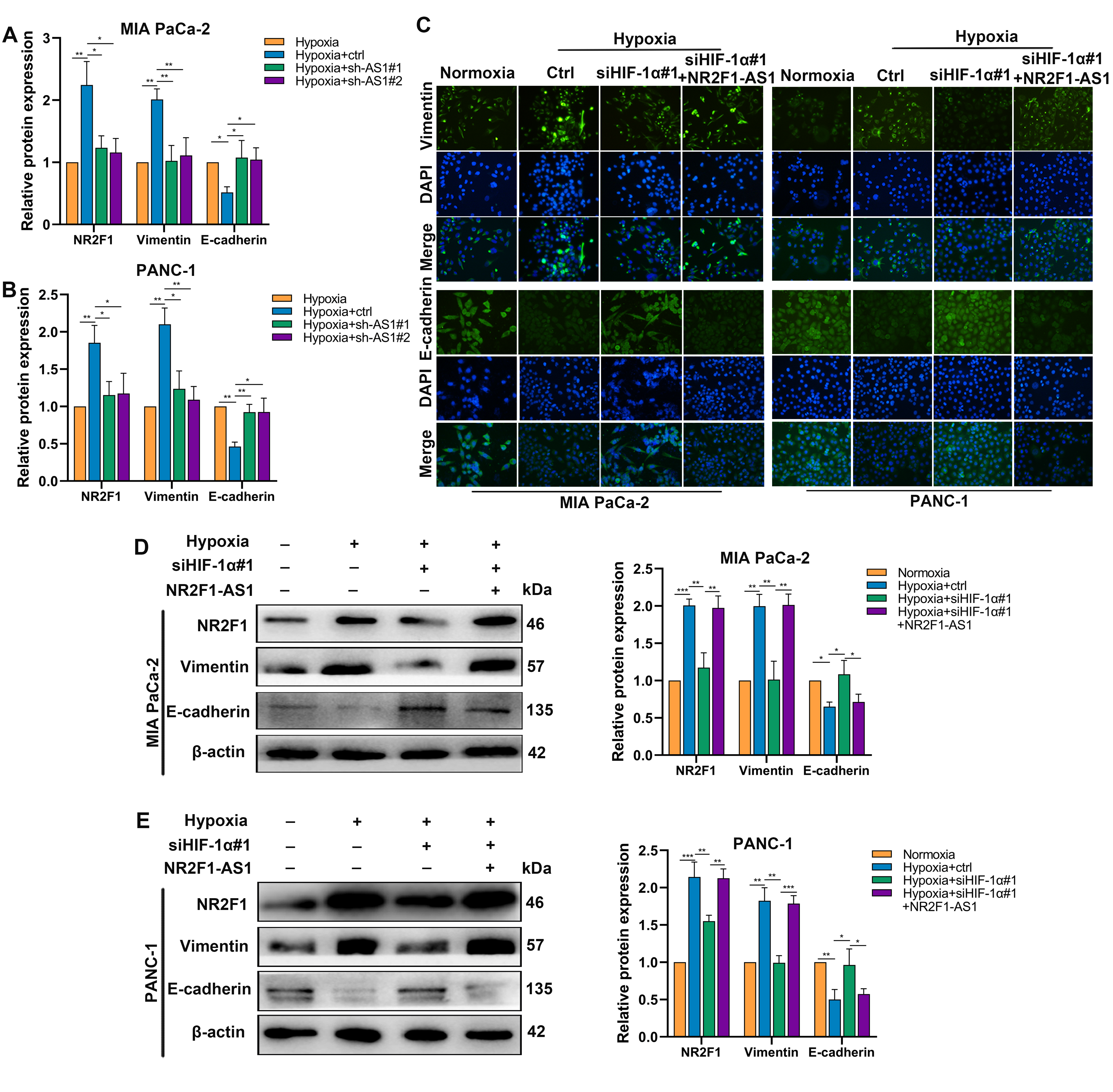


**Supplementary Figure S9. Effect of NR2F1-AS1 on NR2F1, Vimentin, E-cadherin in hypoxia microenvironment of PC cells.**

**A,B** Illustration of NR2F1, Vimentin, and E-cadherin proteins quantification in different treatment groups were detected by western blot in **Fig. 8B. C** IF staining analysis of Vimentin and E-cadherin in different treatment PC cells, Scale bar = 100μm. **D,E** The expression levels of NR2F1, Vimentin and E-cadherin proteins by western blot in corresponding MIA PaCa-2 and PANC-1 cells. Data were expressed as mean ± SD. All experiments were repeated three times independently. **P*<0.05, ***P*<0.01,****P*<0.001.

**Supplementary Figure S10**


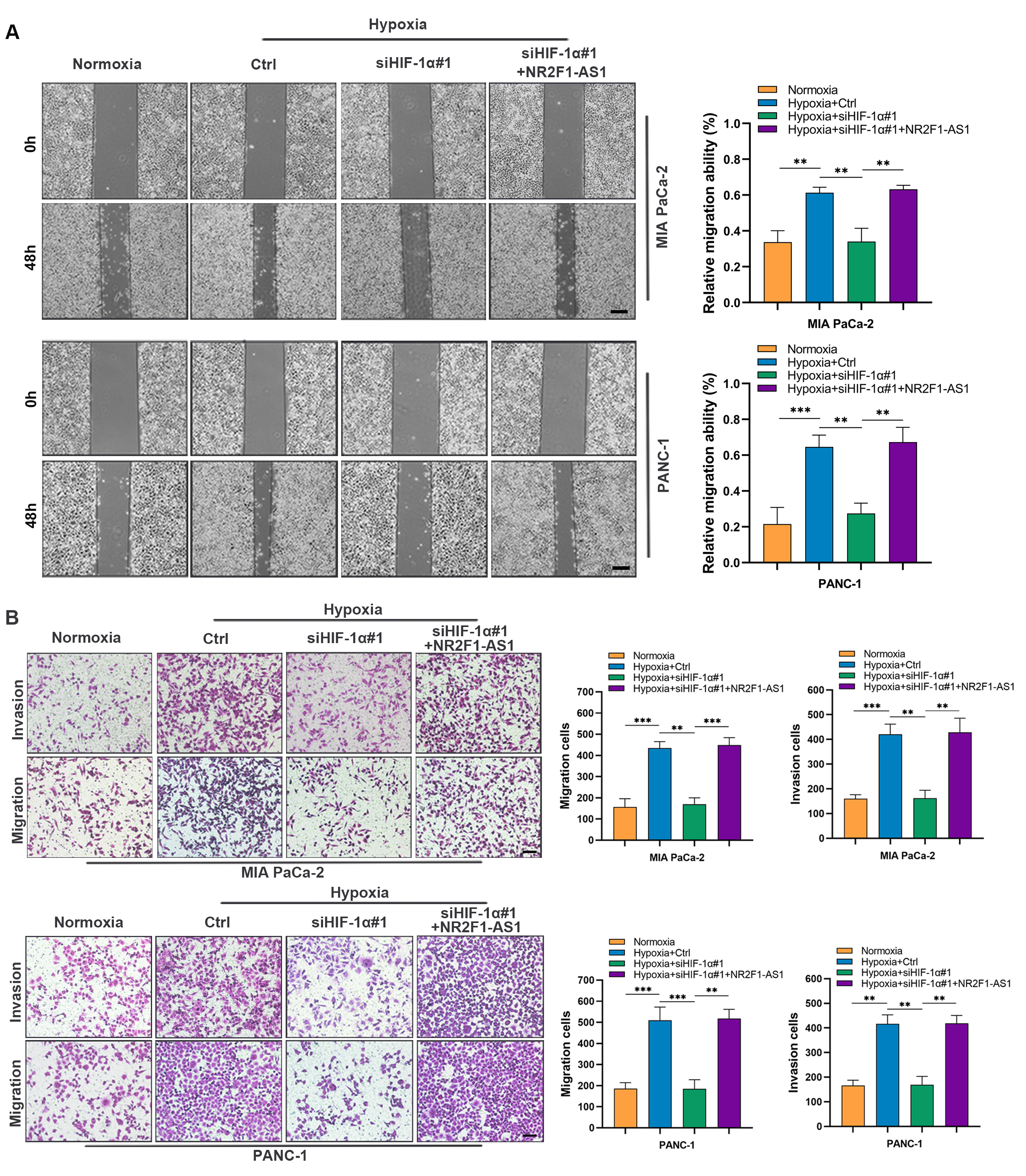


**Supplementary Figure S10. Effect of NR2F1-AS1 on hypoxia-induced migration and invasion in PC cells.**

**A, B** Wound healing and transwell assays were used to detect the migration and invasion ability in different treatment groups in PC cells. Scale bar = 200μm. Data were expressed as mean ± SD. All experiments were repeated three times independently. ***P*<0.01, ****P*<0.001.
