# Supplementary material for: Hypoxia-induced long noncoding RNA NR2F1-AS1 maintains pancreatic cancer proliferation, migration, and invasion by activating the NR2F1/AKT/mTOR axis
Source: Cell Death Dis. 2022 Mar 14;13(3):232. doi: 10.1038/s41419-022-04669-0 (PMC8918554; doi:10.1038/s41419-022-04669-0)
Supplement: Supplementary file 4 — Supplementary Tables [file 41419_2022_4669_MOESM4_ESM.doc]

**Supplementary Table S1. The primer sequences of qRT-PCR.**

| **Genes** |  | **Sequences (5’→3’)** |
| --- | --- | --- |
| NR2F1-AS1 | Forward  Reverse | TCCGTGACCACAATATTAACCA  GAGAACATCTGCTGCAACCTG |
| U6 | Forward  Reverse | CAGCACATATACTAAAATTGGAACG  ACGAATTTGCGTGTCATCC |
| β-actin | Forward  Reverse | CTCCATCCTGGCCTCGCTGT  GCTGTCACCTTCACCGTTCC |
| NR2F1 | Forward  Reverse | TTCGTCCGTTTGGTAGGTAAAA  GAGCACTGGATGGACATGTAAG |
| HIF-1α | Forward  Reverse | AGTTCCGCAAGCCCTGAAAGC  GCAGTGGTAGTAGTAGCATTAGC |
| NR2F1-AS1 | probe | TCCTGGTTAATATTGTGGTCA |

**Supplementary Table S2. Sequences of siRNAs/shRNAs used in this study.**

| **Targets** | **Sequences（5’→3’）** |
| --- | --- |
| sh-NR2F1-AS1#1 | GCTAGATCAGGAAGCCTATG |
| sh-NR2F1-AS1#2 | GAAACTAGCCCATGATGAAC |
| sh-NR2F1-AS1#3 | GGAAGCCTATGTCAACTAT |
| siNR2F1-1 | CCTTACATGTCCATCCAGT |
| siNR2F1-2 | GCAATGGTAGTTAGCAGCT |
| siHIF-1α#1 | GCTGACCAGTTATGATTGT |
| siHIF-1α#2 | CCAGTTACGTTCCTTCGAT |
| siHIF-1α#3 | GATAAGTTCTGAACGTCGA |

**Supplementary Table S3. Antibodies used in this study.**

| **Antibody** | **Catalog number** | **Company** | **Dilution ratio** |
| --- | --- | --- | --- |
| E-Cadherin (24E10) Rabbit mAb | #3195 | Cell Signaling Technology | Western blot:(1:1000)  Immunofluorescence: (1:200) |
| Vimentin (D21H3) XP® Rabbit mAb | #5741 | Cell Signaling Technology | Western blot:(1:1000)  Immunofluorescence: (1:100) |
| Beta Actin Monoclonal Antibody | 66009-1-Ig | Proteintech | Western blot:(1:10000) |
| [Recombinant](https://www.abcam.cn/recombinant-human-coup-tf1-protein-ab152739.html)Anti-COUP TF1antibody [EPR10841] | ab181137 | Abcam | Western blot:(1:1000)  Immunofluorescence: (1:100) |
| Akt (pan) (C67E7) Rabbit mAb | #4691 | Cell Signaling Technology | Western blot:(1:1000) |
| Phospho-Akt (Ser473) (D9E) XP® Rabbit mAb | #4060 | Cell Signaling Technology | Western blot:(1:2000) |
| Phospho-Akt (Thr308) (D25E6) XP® Rabbit mAb | #13038 | Cell Signaling Technology | Western blot:(1:1000) |
| mTOR (7C10) Rabbit mAb | #2983 | Cell Signaling Technology | Western blot:(1:1000) |
| [Phospho-mTOR (Ser2448) (D9C2) XP® Rabbit mAb](https://www.cellsignal.cn/products/primary-antibodies/phospho-mtor-ser2448-d9c2-xp-rabbit-mab/5536?site-search-type=Products&N=4294956287&Ntt=p-mtor&fromPage=plp) | #5536 | Cell Signaling Technology | Western blot:(1:1000) |
| Phospho-p70 S6 Kinase (Thr389) Antibody | #9205 | Cell Signaling Technology | Western blot:(1:1000) |
| HIF-1α Rabbit Polyclonal Antibody | 20960-1-AP | Proteintech | Western blot:(1:5000) |
| PCNA Rabbit Polyclonal Antibody | 10205-2-AP | Proteintech | Immunohistochemistry:(1:200) |
| Rabbit Rabbit anti-human Ki67 | 27309-1-AP | Proteintech | Immunohistochemistry:(1:2000) |
| HRP Conjugated AffiniPure Goat Anti-mouse IgG (H+L) | BA1051 | Boster Biological Technology | Western blot:(1:5000) |
| HRP Conjugated AffiniPure Goat Anti-rabbit IgG (H+L) | BA1055 | Boster Biological Technology | Western blot:(1:5000) |
| FITC Conjugated AffiniPure Goat Anti-Rabbit IgG (H+L) | BA1105 | Boster Biological Technology | Immunofluorescence: (1:100) |
| Alexa Fluor® 488-conjugated Goat Anti-Rabbit IgG (H+L) | GB25303 | servicebio | Immunofluorescence: (1:200) |

**Supplementary Table S4 Correlation between NR2F1-AS1 expression and the clinicopathological features of pancreatic cancer.**

| **Clinicopathologic**  **features** | | | **NR2F1-AS1 Expression** | | --- | | | ***p* value** |
| --- | --- | --- | --- | --- |
|  |  | **Low (n)** | **High (n)** |  |
| All cases |  | 32 | 42 |  |
| Age (years) |  |  |  |  |
|  | <60 | 9 | 19 | 0.1326 |
|  | ≥60 | 23 | 23 |  |
| Gender |  |  |  |  |
|  | Male | 17 | 30 | 0.1051 |
|  | Female | 15 | 12 |  |
| TNM stage |  |  |  |  |
|  | I and II | 29 | 37 | 0.7284 |
|  | III and IV | 3 | 5 |  |
| Lymph node metastasis |  |  |  |  |
|  | N0 | 23 | 26 | 0.3690 |
|  | N1 | 9 | 16 |  |
| Nerve invasion |  |  |  |  |
|  | Negative | 21 | 17 | **0.0320^*^** |
|  | Positive | 11 | 25 |  |
| Diameter of tumor(cm) |  |  |  |  |
|  | <3 | 11 | 6 | **0.0418^*^** |
|  | ≥3 | 21 | 36 |  |
| Pathological grading |  |  |  |  |
|  | I | 2 | 2 | 0.9590 |
|  | II | 19 | 25 |  |
|  | III | 11 | 15 |  |
