# Supplementary figures and images for: Hypoxia-induced long noncoding RNA NR2F1-AS1 maintains pancreatic cancer proliferation, migration, and invasion by activating the NR2F1/AKT/mTOR axis
Source: Cell Death Dis. 2022 Mar 14;13(3):232. doi: 10.1038/s41419-022-04669-0 (PMC8918554; doi:10.1038/s41419-022-04669-0)

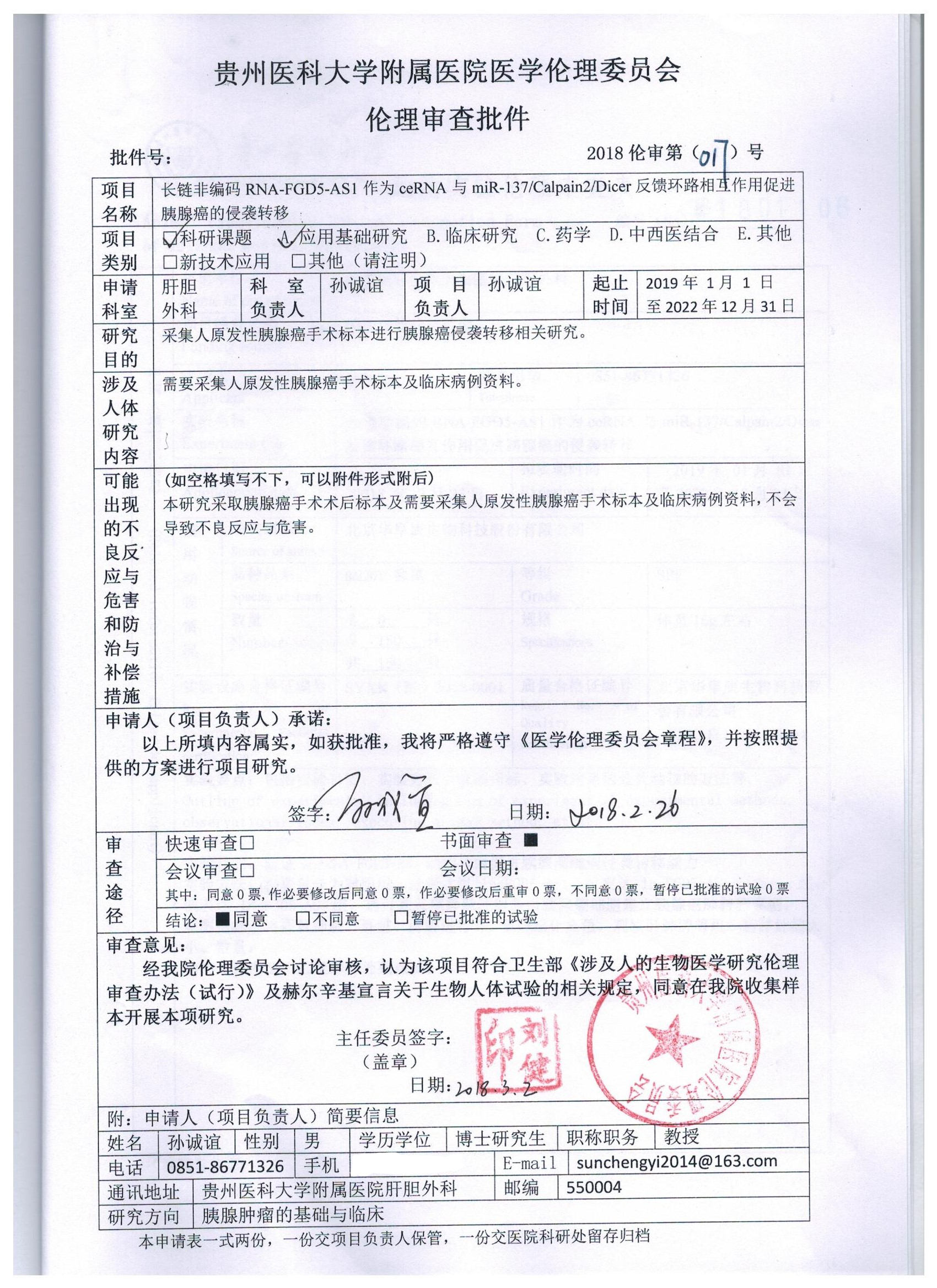

Supplement: Supplementary file 7 — IRB approval [file 41419_2022_4669_MOESM7_ESM.tif]
