# Supplementary material for: Hypoxia-induced long noncoding RNA NR2F1-AS1 maintains pancreatic cancer proliferation, migration, and invasion by activating the NR2F1/AKT/mTOR axis
Source: Cell Death Dis. 2022 Mar 14;13(3):232. doi: 10.1038/s41419-022-04669-0 (PMC8918554; doi:10.1038/s41419-022-04669-0)
Supplement: Supplementary file 8 — The original data of WB blots [file 41419_2022_4669_MOESM8_ESM.pdf]

FIG.2F

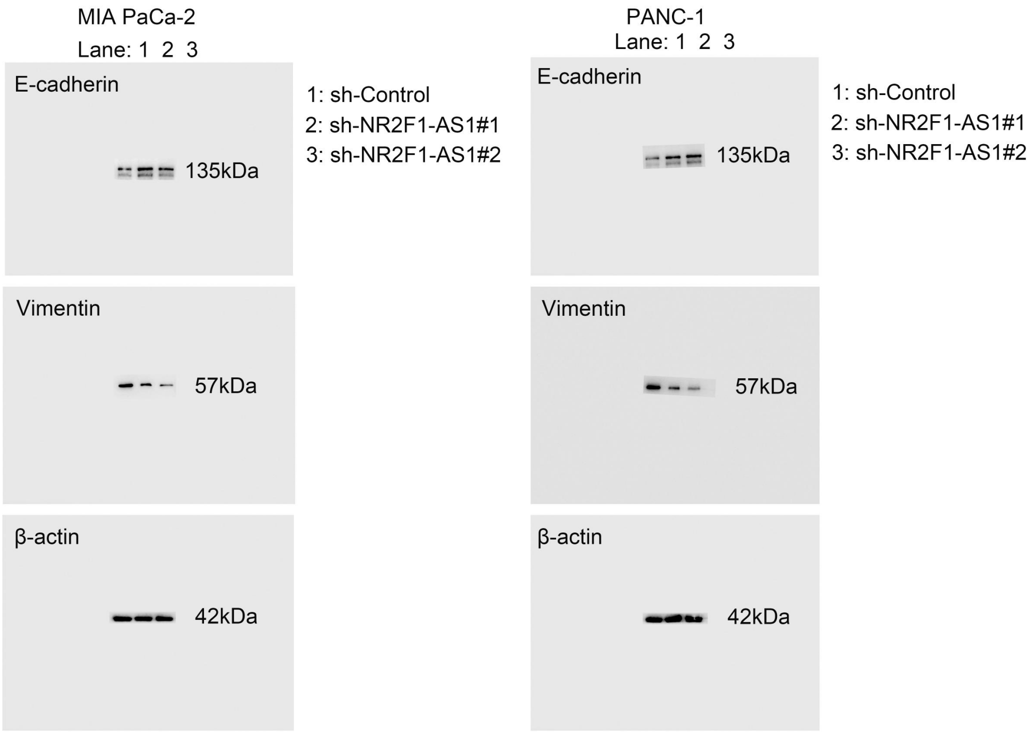

FIG.4F

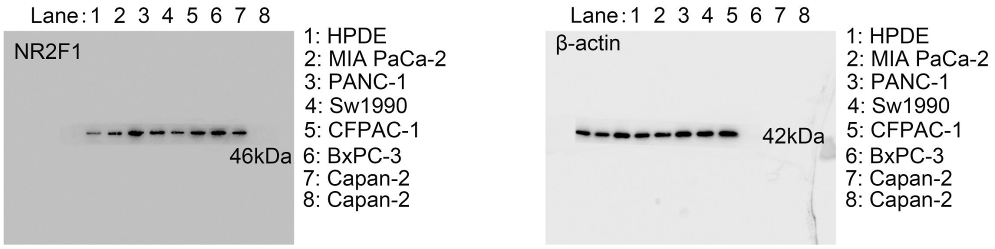

FIG.4J

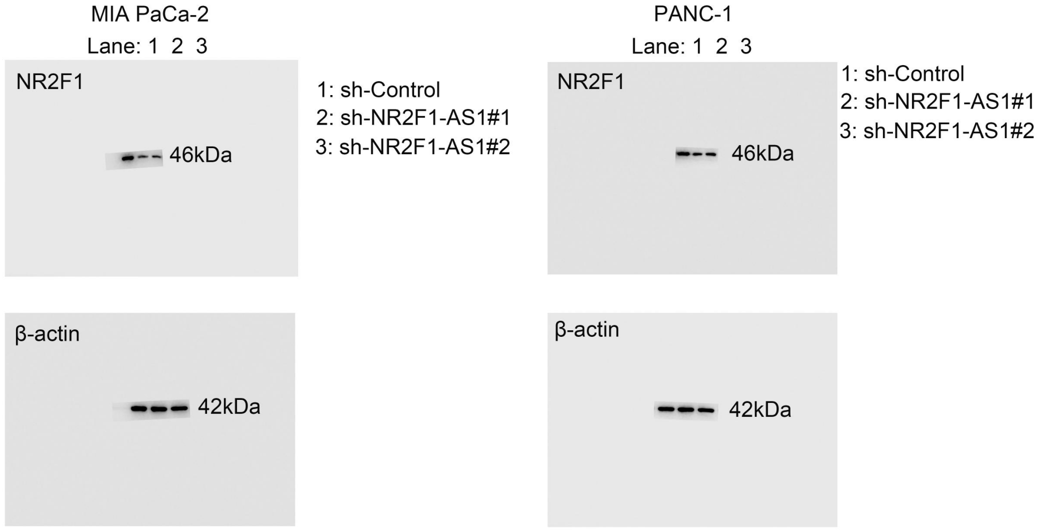

FIG.6B MIA PaCa-2

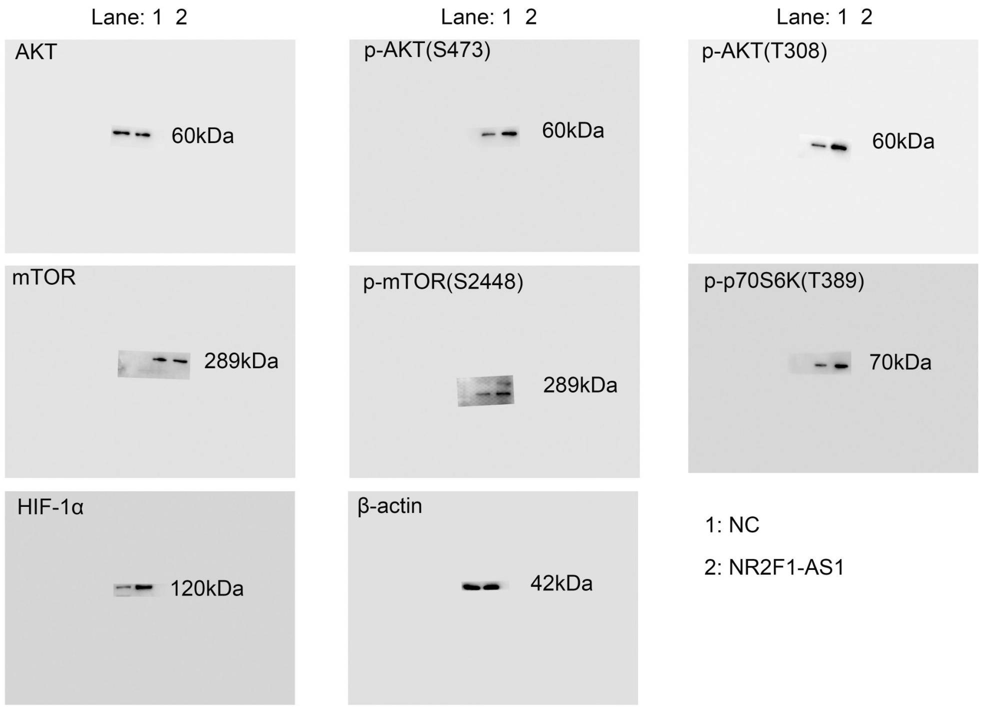

FIG.6B MIA PaCa-2

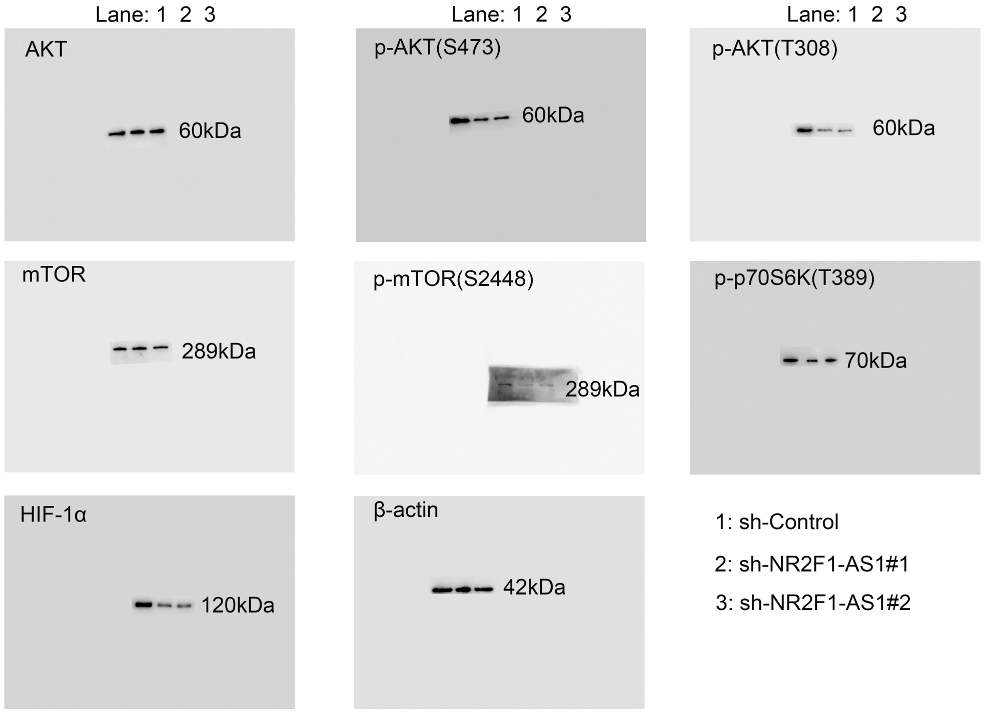

FIG.6B PANC-1

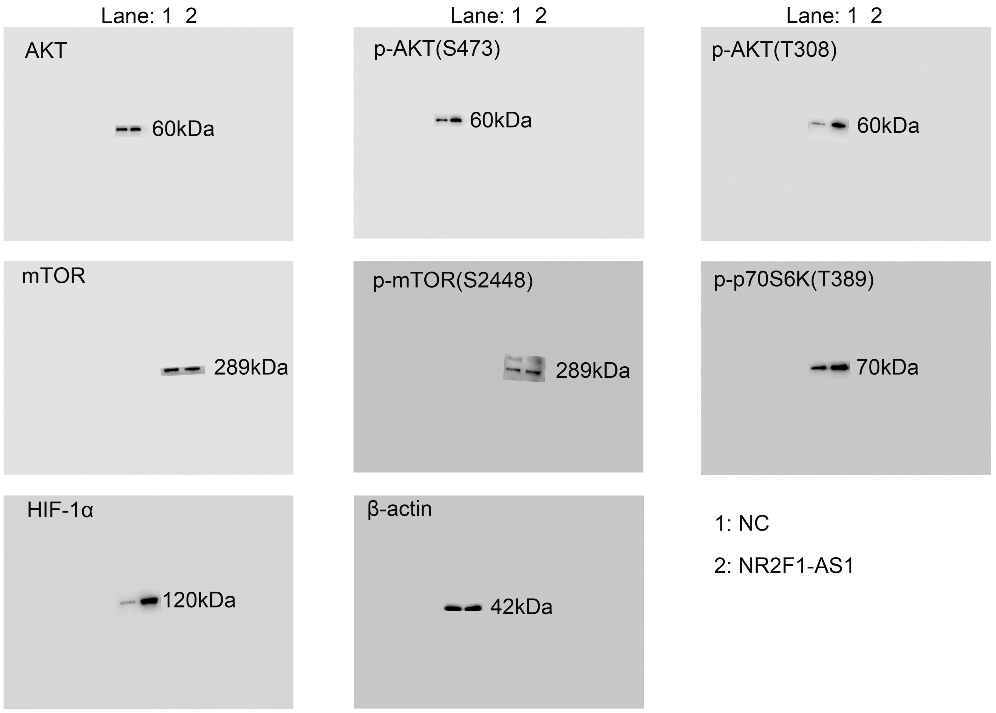

FIG.6B PANC-1

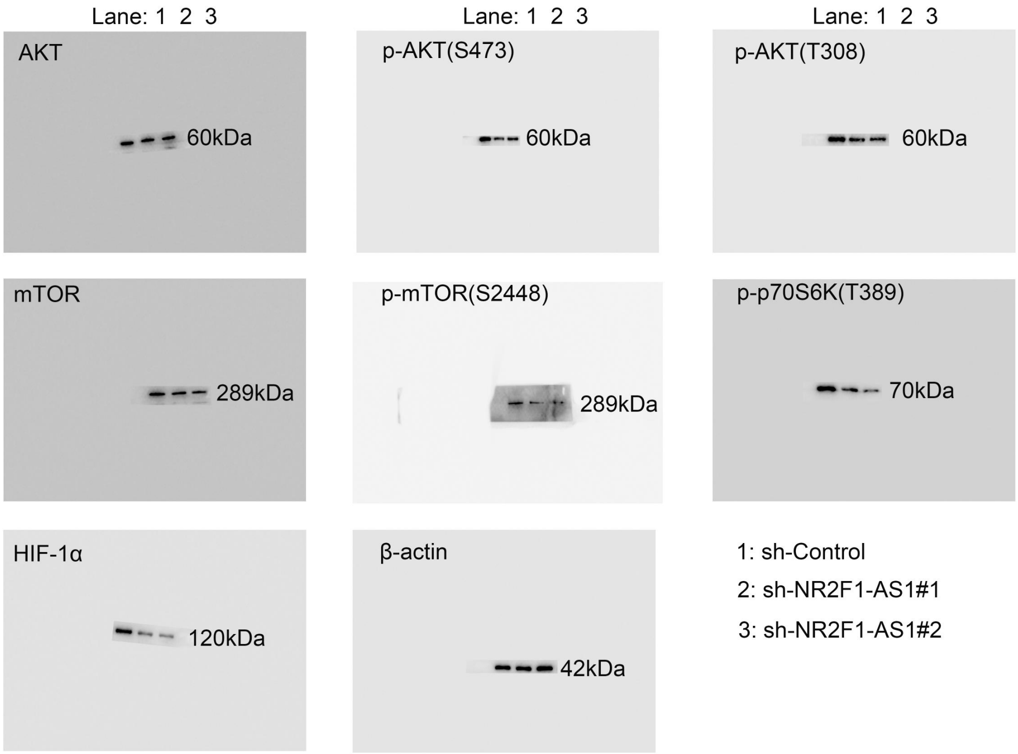

FIG.6G MIA PaCa-2

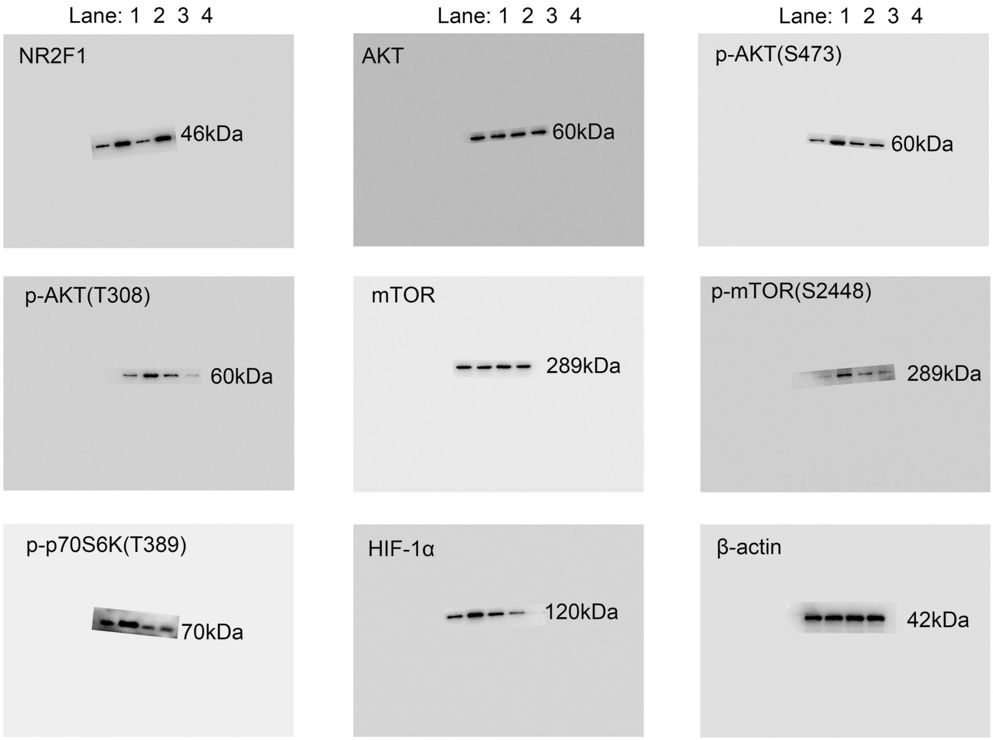

1: NC 2: NR2F1-AS1 3: NR2F1-AS1+siNR2F1 4: NR2F1-AS1+LY294002

FIG.6G PANC-1

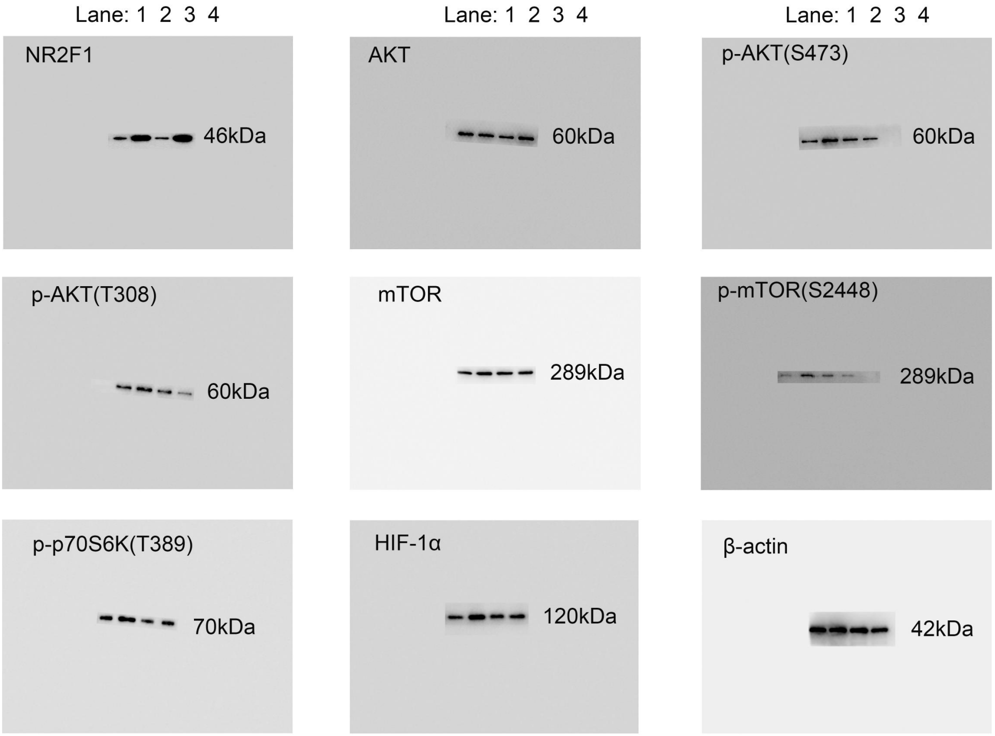

1: NC 2: NR2F1-AS1 3: NR2F1-AS1+siNR2F1 4: NR2F1-AS1+LY294002

FIG.7E

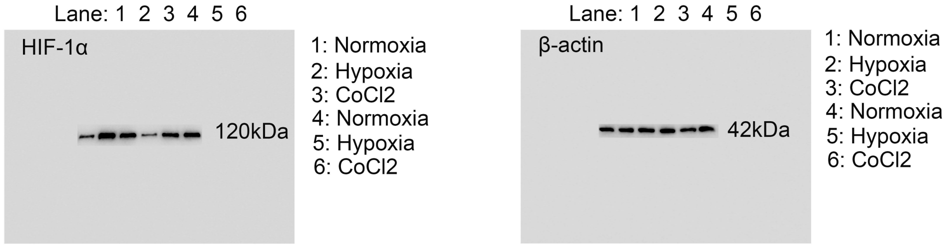

FIG.7H MIA PaCa-2

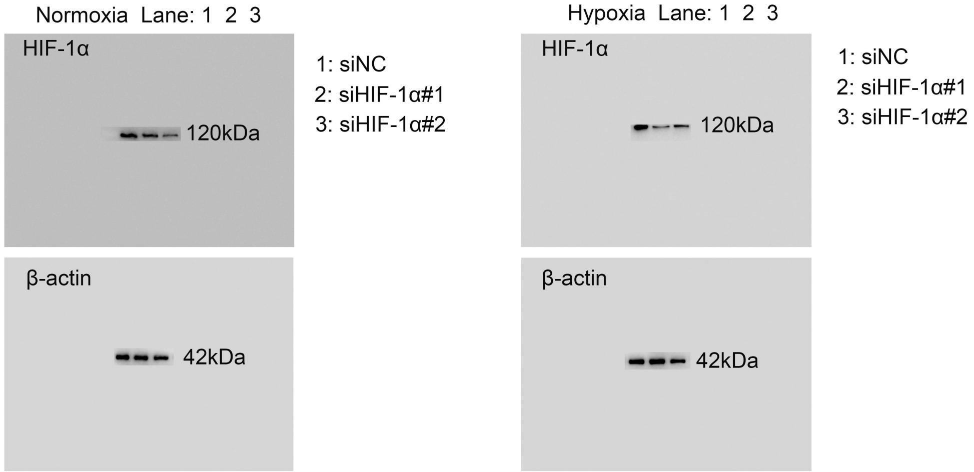

FIG.7H PANC-1

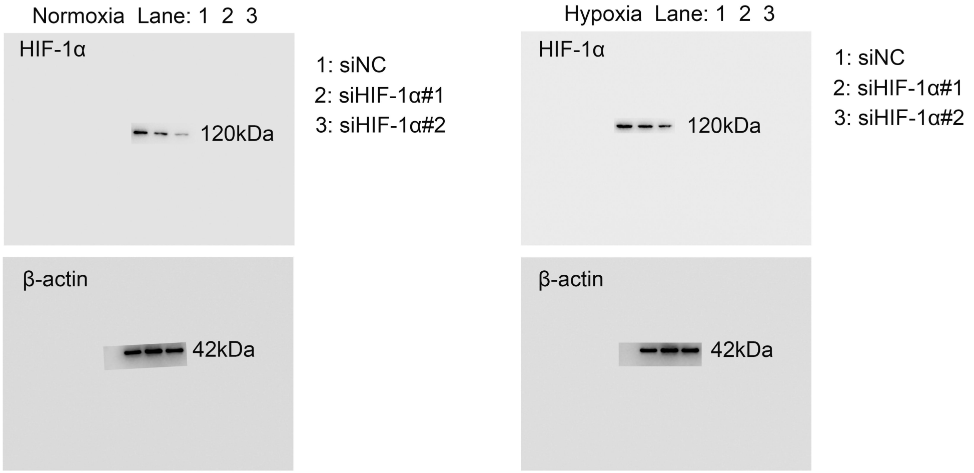

FIG.8B

MIA PaCa-2

Lane: 1 2 3 4

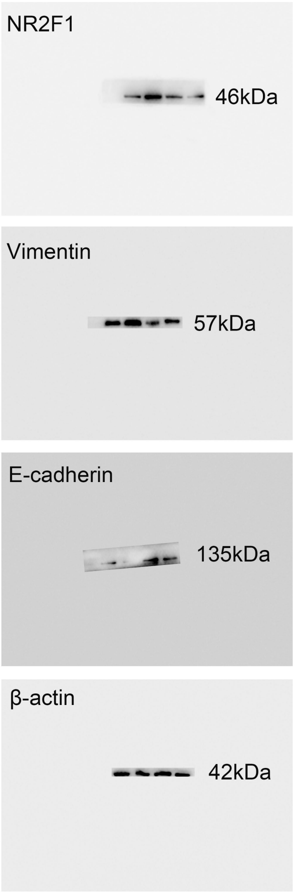

1: Normoxia
2: Hypoxia+sh-Control
3: Hypoxia+sh-NR2F1-AS1#1
4: Hypoxia+sh-NR2F1-AS1#2

PANC-1

Lane: 1 2 3 4

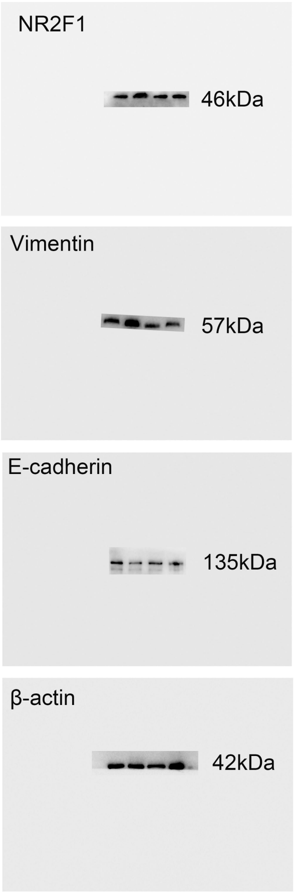

1: Normoxia
2: Hypoxia+sh-Control
3: Hypoxia+sh-NR2F1-AS1#1
4: Hypoxia+sh-NR2F1-AS1#2

Supplementary Figure S6

MIA PaCa-2

Lane: 1 2 3

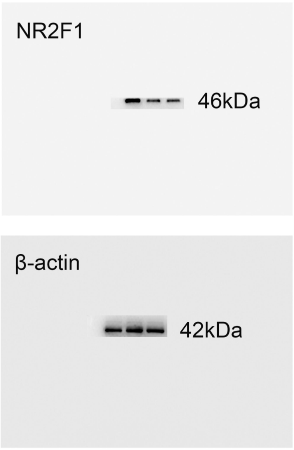

1: siNC
2: siNR2F1-1
3: siNR2F1-2

PANC-1

Lane: 1 2 3

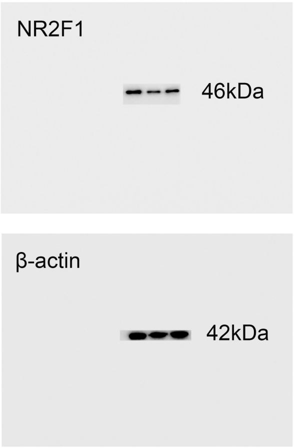

1: siNC
2: siNR2F1-1
3: siNR2F1-2

Supplementary Figure S8

Lane: 1 2 3 4

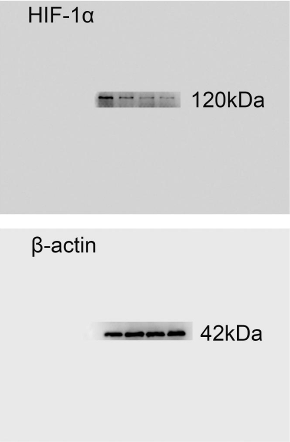

1: siNC
2: siHIF-1α#1
3: siHIF-1α#2
4: siHIF-1α#3

Lane: 1 2 3 4

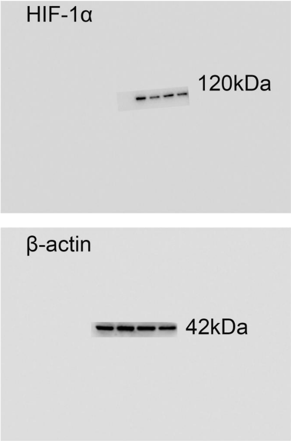

1: siNC
2: siHIF-1α#1
3: siHIF-1α#2
4: siHIF-1α#3

Supplementary Figure S9

MIA PaCa-2

Lane: 1 2 3 4

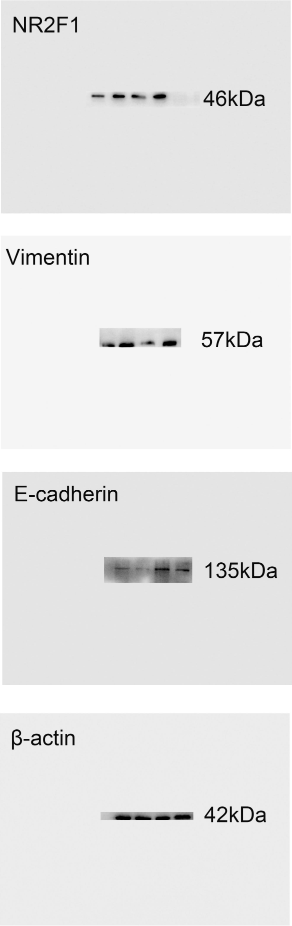

1: Normoxia
2: Hypoxia+ctrl
3: Hypoxia+siHIF-1α#1
4: Hypoxia+siHIF-1α#1+NR2F1-AS1

PANC-1

Lane: 1 2 3 4

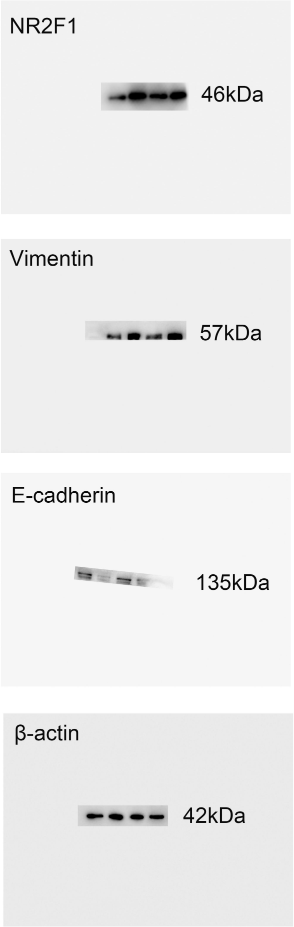

1: Normoxia
2: Hypoxia+ctrl
3: Hypoxia+siHIF-1α#1
4: Hypoxia+siHIF-1α#1+NR2F1-AS1
